# Dissecting the genetic basis of yield stability in faba bean by multi-environment analysis

**DOI:** 10.1186/s13059-026-04181-0

**Published:** 2026-07-20

**Authors:** Elesandro Bornhofen, Troels W. Mouritzen, Sheila Alves, Thomas Ramsay Robertson-Shersby-Harvie, Cathrine Kiel Skovbjerg, Alex Windhorst, Hailin Zhang, Thilani Bhagya Jayakody, Jing Zhang, Marcin Nadzieja, Hyeonah Shim, Jean-Bernard Magnin-Robert, Grégoire Aubert, Matthieu Floriot, Camille Guiziou, Olaf Sass, Gregor Welna, Ignacio Solís, Linda Kærgaard Nielsen, Natalia Gutiérrez, Murukarthick Jayakodi, Frederick L. Stoddard, Donal Martin O’Sullivan, Ana M. Torres, Wolfgang Link, Nadim Tayeh, Luc Janss, Stig Uggerhøj Andersen

**Affiliations:** 1 Center for Quantitative Genetics and Genomics, Aarhus University, Aarhus, Denmark; 2 Department of Molecular Biology and Genetics, Aarhus University, Aarhus, Denmark; 3 Teagasc, Crops Research, Oak Park, Carlow, Ireland; 4 School of Agriculture, Policy and Development, University of Reading, Reading, UK; 5 Nordic Seed A/S, Odder, Denmark; 6 Division of Plant Breeding Methodology, Georg-August-University Goettingen, Goettingen, Germany; 7 Leibniz Institute of Plant Genetics and Crop Plant Research (IPK) Gatersleben, Seeland, Germany; 8 Université Bourgogne Europe, Institut Agro Dijon, INRAE, Agroécologie, Dijon, France; 9 Agri Obtentions, Guyancourt, France; 10 Norddeutsche Pflanzenzucht Hans-Georg Lembke KG, Holtsee, Germany; 11 Agrovegetal, Seville, Spain; 12 Sejet Plant Breeding, Horsens, Denmark; 13 Área de Mejora Vegetal y Biotecnología, IFAPA, Córdoba, Spain; 14 Department of Soil and Crop Sciences, Texas A&M AgriLife Research Dallas Center, Dallas, TX USA; 15 Department of Agricultural Sciences, University of Helsinki, Helsinki, Finland

## Abstract

**Background:**

Faba bean is a globally adapted legume protein crop with a high yield potential. Currently, yield variation across environments limits more widespread cultivation, and the underlying genetics remain poorly understood.

**Results:**

Here, we identify major QTL for faba bean yield and yield stability. We genotype the ProFaba diversity panel with high resolution and carry out coordinated multi-year/location trials across Europe. Based on these data, we identify more than one hundred loci associated with mean performance and stability for 14 complex traits, including yield. Experimental validation supports the involvement of the candidate gene *Vfaba.Hedin2.R2.1g002122* in plant architecture, with gene expression significantly associated with first pod position and plant height. Furthermore, we introduce a method for integrating environmental data in the analysis of trait stability based on a random regression mixed model, which enables prediction of performance in untested environments.

**Conclusions:**

Our study provides insights into the genetic architecture of yield, yield stability, and genotype-by-environment interaction in faba bean. The genomic resources, candidate loci, and weather-informed analytical framework provide practical tools for predicting performance across environments and accelerating breeding of resilient, high-yielding protein crops.

**Supplementary Information:**

The online version contains supplementary material available at 10.1186/s13059-026-04181-0.

## Background

Breeding climate-resilient crops to withstand the adverse conditions associated with increasing climate instability is crucial for securing future food supplies. Furthermore, the demand for locally produced sources of macronutrients is on the rise, which oftentimes implies pushing crops to the limits of geographical adaptation. One such crop is faba bean (*Vicia faba* L., 2n = 12), a globally adapted legume protein crop that has gained attention for its high yield potential [1], high protein content (approximately 29%), and ability to efficiently fix atmospheric N_2_. It is an annual cool-season crop with adaptation spanning production zones in distinct growing regions around the globe. Despite its attractive characteristics, yield stability remains an unresolved limiting factor, hampering acreage expansion and potential benefits within sustainable cropping systems. Drought sensitivity is often pointed out as one of the main reasons for spatiotemporal variation in faba bean yield, but other factors such as heat and cold stress [2] also contribute to the observed yield instability. Genetic variability has been reported for tolerance to major stressors [3, 4], which contribute to differential performance of genotypes for key agronomic traits as plants are exposed to variable environmental conditions, a phenomenon known as genotype-by-environment interaction (GxE). Large-scale studies of GxE in faba beans are necessary to shed light on biological mechanisms and factors controlling phenotypic plasticity and trait stability.

Phenotypic plasticity refers to the ability of an individual organism to express different phenotypes in response to changes in environmental conditions [5]. Measuring plasticity requires exposing genotypes to perturbations due to distinct environmental conditions and analyzing the data using appropriate statistical methods. A widely used approach is to estimate reaction norms of genotypes as a function of an environmental gradient metric, typically environmental means (average performance of all tested genotypes at each environment), although any continuous environmental descriptor can be utilized, which may allow predicting genotypic performance in untested conditions [6, 7]. In reaction norm models, mean performance and sensitivity estimates are readily obtained from solutions of intercept and slope, respectively. Variations in these two quantities can be mapped to genomic regions via genome-wide association studies (GWAS) [8, 9], revealing single-nucleotide variants for marker-assisted selection in breeding programs and candidate genes for genetic studies. Even in the absence of large-effect QTL for highly polygenic traits, faba bean breeders may still benefit from incorporating stability into the genomic evaluation system, provided heritability is present.

Interaction models represent another approach for mapping genomic regions associated with environment-specific responses of quantitative traits [10]. Such models can identify QTL exhibiting antagonistic pleiotropy, characterized by sign-changing additive effects; conditional neutrality, where effects are confined to specific environmental conditions; and differential sensitivity, which results from varying magnitudes of additive effects across different environments [11]. These modeling strategies are complementary and can help uncover the biological mechanisms underlying yield stability or plastic responses to varying environmental conditions across multi-environment trials spanning large geographical areas. Breeders can apply this knowledge to develop stable varieties by selecting against alleles known to disrupt stability or by designing genotypes tailored to specific growing regions, utilizing environment-specific beneficial QTL.

Here, we present extensive multi-year/location field trials and high-resolution genotype data for the ProFaba panel [12]. We combine the data to elucidate the genetic architecture underlying mean performance and GxE across a range of complex traits in faba bean, including seed yield, and demonstrate large-scale prediction of genotypic performance across unseen environments.

## Results

### The ProFaba panel

**Fig. 1:**
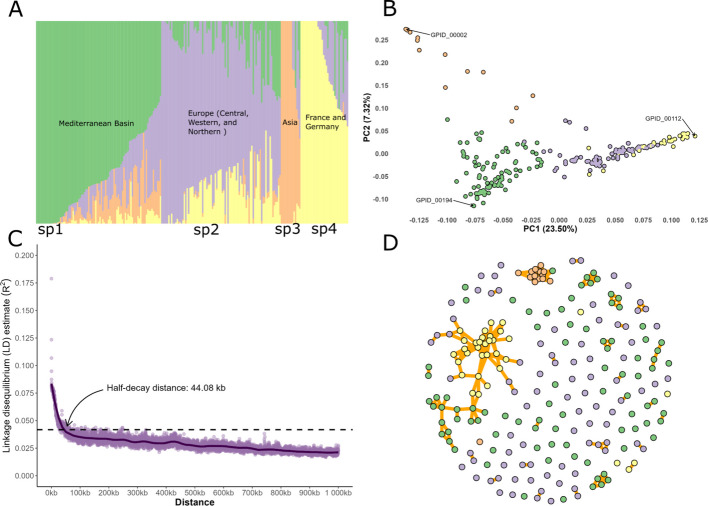
Four faba bean subpopulations identified by admixture analysis. **A** Genetic ancestry composition for each of the 222 accessions composing the ProFaba panel for a value of K = 4 (four sub-populations: sp1 to sp4) revealed by cross-validation using ADMIXTURE. **B** Principal component analysis depicting accessions colored by subpopulation membership according to the ADMIXTURE results. **C** Linkage disequilibrium (LD; pairwise squared correlation) as a function of the physical distance in kb between SNPs. **D** Genomic relationship matrix (GRM) shown as a network where each node represents each of the 222 accessions and edges are connecting nodes for which the genomic correlation is ≥ 0.2, representing a cutoff on the 99th percentile. Nodes of the network were placed in the plane using the Fruchterman-Reingold layout algorithm, and are also colored according to the results in **A**

The ProFaba diversity panel consists of a global collection of 234 faba bean lines. It includes both spring and winter European types, along with accessions from North Africa, Latin America, the Middle East, the Far East, and Australia. The panel also covers all three seed size classes: minor, equina, and major type. A detailed description of the constituents of the panel is provided in Skovbjerg et al. [12]. We genotyped the panel using genotyping-by-sequencing (GBS) [13] and single primer enrichment technology (SPET) [14], which yielded 540 K high-quality SNP markers, of which 347 K were validated using 10-fold whole genome resequencing (WGS) data. A concordance rate of 98.3% was verified between SPET + GBS and WGS datasets using the reference genotype Hedin/2 present in both. Variants are well distributed across the genome (Additional file 1: Fig. S1) and are of an average density of 53 variants per Mb. The average proportion of homozygous loci across lines was calculated to be 95.5%. We analyzed the genotype data using ADMIXTURE [15] and found four subpopulations represented in the diversity panel (Fig. 1A). Accessions within subpopulation sp1 predominantly trace their origins to the Mediterranean Basin, whereas those in sp2 primarily derive from Central, Western, and Northern Europe. For the two smaller subpopulations, sp3 represents accessions from Asia while lines from France and Germany are represented in sp4. The result of the ADMIXTURE analysis was used to color accessions in the PCA plot and for the genomic correlation network (Fig. 1B and D). Plotting accessions according to the first two eigenvectors revealed a clear separation of subpopulations, albeit with marginal overlap between sp2 and sp3. Moreover, members within sp3 and sp4 exhibited higher relatedness, as indicated by the network's node connectivity, with edges representing a Pearson correlation of ≥ 0.2 (Fig. 1D).

The estimation of linkage disequilibrium based on $R^2$ values revealed a decay to half of its maximum value at 44.08 kb distance (Fig. 1C). For SNPs within the first bin (1–101 bp), the average distance was 44.61 bp and the average $R^2$ was 0.179. The genome-wide nucleotide diversity (π) was 0.25. Values of π per subpopulation were: 0.24 for sp1 and sp2, 0.19 for sp3, and 0.23 for sp4, indicating slightly lower genetic diversity in sp3 compared to the others. The average proportion of heterozygosity was 4.48% across the whole panel, and the average minor allele frequency was 16.73%. The diversity represented in this collection of faba bean accessions makes it well-suited for genetic parameter estimation and gene discovery.

The ProFaba diversity panel was evaluated in field conditions through a collaborative effort between public and private partners (Additional file 2: Table S5). Field trials were conducted in Denmark, Ireland, France, Germany, and Spain from 2019 to 2021. A total of 48 traits were assessed, including seed yield, morphological traits, and traits related to biotic stress. The curated data can be found at Zenodo [16]. In this study, we analyzed data for 14 traits (Additional file 2: Table S6) across 12 environments (year-location combinations), with some traits missing in certain environments (Additional file 1: Fig. S2). This set of environments covering distinct weather and soil features was essential for investigating faba bean GxE interactions. Adjusted line means across traits are available in Additional file 1: Table S7. A considerable phenotypic variation is observed across lines for traits such as seed yield (coefficient of variation of 39%), branch number per plant (33%), and rust (33%).

### Unsupervised learning of environmental relationships

**Fig. 2:**
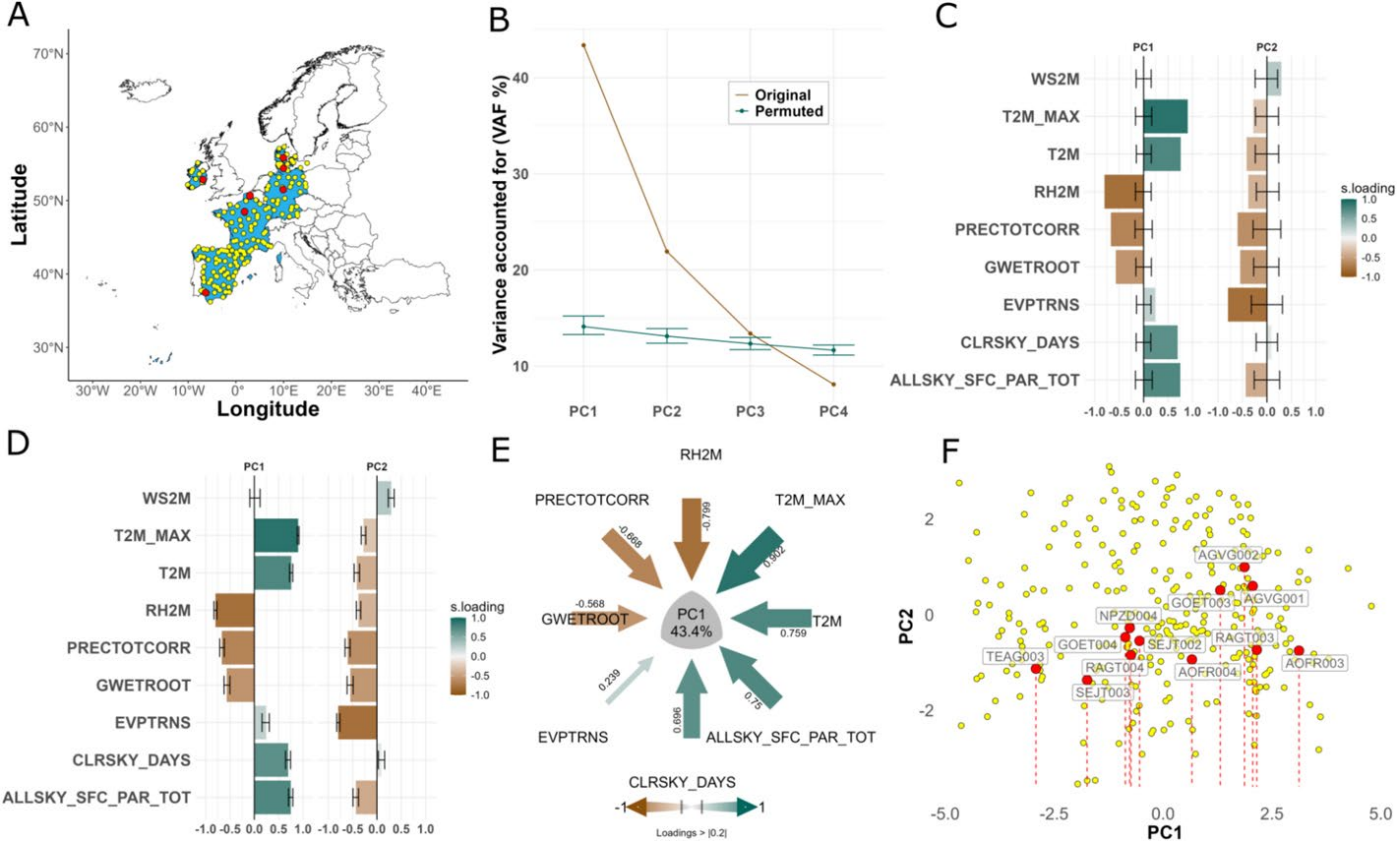
Constructing a weather-derived covariate for reaction norm modeling. **A** Map of Europe with countries where field trials were conducted are highlighted in blue. Points in red refer to field trial locations. Points in yellow denote 299 randomly sampled coordinates for which weather data was extracted and submitted to principal component analysis. **B** line plot showing the variance explained by the first four PCs computed with the original and permuted variables. **C** principal component identity obtained by permutation test for loadings. Error bars denote mean ± 95% confidence interval (CI) of permuted samples. **D** component stability and ± 95% CI by bootstrapping. **E** syndromic plot showing the Pearson correlation of each weather variable with the first principal component. **F** projection of the true sites (points in red) onto the multidimensional space learned from weather data of random locations (points in yellow). T2M, averages of mean temperature; T2M_MAX, mean maximum temperature; GWETROOT, root zone soil wetness; PRECTOTCORR, precipitation corrected; EVPTRNS, evapotranspiration energy flux; WS2M, wind speed; RH2M, relative humidity; ALLSKY_SFC_PAR_TOT, photosynthetically active radiation; and CLRSKY_DAYS, clear sky day

One way to assess GxE is through reaction norm models, which are traditionally fitted in two steps. Recent advances have improved upon the traditional two-step approach by simultaneously inferring environmental means, treated as an unknown covariate, along with other model parameters within a Bayesian framework [17]. However, interpretations are limited as environmental means are a result of numerous drivers and predictions can not be drawn for untested environments. An alternative approach is to explicitly regress on known environmental covariates or their linear combinations [6]. Here, we introduce a method for constructing synthetic covariates using eigenvalue decomposition of environmental variables collected from randomly selected sites across broad geographical areas (Fig. 2A). Significant principal components capturing most of the variance in the dataset (Fig. 2B) are characterized via standardized loadings of each environmental variable (Fig. 2C), which are also checked for stability by bootstrapping (Fig. 2D). Next, correlations of significant variables with principal component scores are computed, providing an identity to each component (Fig. 2E). Finally, the true environments, where field trials were conducted, are projected into the formed multidimensional space and synthetic covariates are extracted as the scores of these environments on significant principal components (Fig. 2F). We utilized the scores of the first principal component, which explained 43.4% of the variance, as a synthetic covariate, referred to as “synCov” throughout the text. The slopes derived from a reaction norm model using synCov were analyzed through genome-wide association studies. Since synCov captures a gradient of environments ranging from cold, humid, and cloudy to warm, sunny, and dry, we aimed to identify QTL that explain variation in slopes driven by this set of environmental descriptors.

### Variance explained by genetic factors

We applied two reaction norm models to the data: one using an unknown covariate (unkCov) [17] and the other with our synthetic covariate (synCov). We extracted the solutions for the intercept and slope in the first model and the slopes in the second model. We investigated the proportion of phenotypic variance explained by all SNP markers (PVE) and the extent of it that is due to markers with major effect, also referred to as sparse effects (PGE) (Additional file 3: Table 1). This offers an insight on the polygenicity of the trait, revealing the importance of minor effect QTL spreading the heritability across several loci. We estimated PVE, PGE, and an approximate number of SNPs showing sparse effects by fitting a Bayesian sparse linear mixed model (BSLMM) to the data [18]. Estimates of PVE ranged from 0.70 to 0.99 for the intercept, while estimates of PGE varied from 0.21 to 0.83. Our findings suggest a sparse genetic architecture for traits related to flowering and plant height, with major-effect loci making a moderate contribution to the mean performance of seed yield. Regarding seed yield stability, the results indicate an important role of sparse effects and, therefore, a simpler architecture, with nearly 50 large-effect SNPs accounting for over half of the phenotypic variance explained by all available genotypes. Estimates for the exact number of SNPs with sparse effect are not very precise (high posterior standard deviation), probably due to the limited sample size. Additionally, estimates of intercepts and slopes for traits measured in a limited number of environments may be less precise due to the strong influence of local environmental factors.

Estimates of intercept and slope variances and covariance obtained from reaction norm models were used to calculate the genetic correlation and genetic variance along the environmental gradient defined by the unknown and the synthetic covariates. We identified substantial GxE interaction using the unknown covariate for rust, vigor, seed yield, pod number per node, pod length, and seed number per pod (Additional file 1: Fig. S3) with SNP heritability ranging from 0.63 to 0.95 and moderate to small contribution of sparse effects (Additional file 3: Table 1). These were essentially the same traits with the highest GxE interaction computed from the reaction norm model using the synthetic covariate (Additional file 1: Fig. S4). For the remaining traits, GxE was limited, and consequently, estimates of PVE for slopes were small with larger uncertainties.

We observed large changes in genetic variances as a function of the environmental covariate (Additional file 1: Figs. S5 and S6), especially for estimates produced by the reaction norm model using an unknown covariate. Such changes in genetic variance contribute to a form of interaction known as scale-type GxE, which has no effect on the rank of genotypic values across environments. Variation in reaction norm slopes that are solely due to rank-type GxE can be obtained by an orthogonalization procedure named genetic regression [19, 20]. The regression produces a corrected slope that is adjusted to be independent of the intercept, ensuring that it specifically reflects the re-ranking component of the GxE interaction. We implemented this method in our analysis and the resulting scale-corrected reaction norms are depicted in Additional file 3: Fig. 1. Cross-over interaction along the environmental gradient can be observed for most traits and for both versions of the reaction norm model. Additionally, the color grouping factor for reaction norms not only allows inference of overall performance according to the genetic background of subpopulations, as depicted in Fig. 1A, but also how they respond to environmental improvement (slope unkCov) or changes in overall weather patterns across the tested population of environments (slope synCov). For seed yield, accessions belonging to sp1 (southern Europe) showed positive slopes (synCov) as the environment shifted from cold and humid to warm and dry. The opposite was observed for accessions from sp2 (Central, Western, and Northern Europe) and sp3 (Asia). This is in line with the geographical origin of the component accessions of these subpopulations.

### Single-stage genome-wide scan of QTLxE interaction

**Fig. 3:**
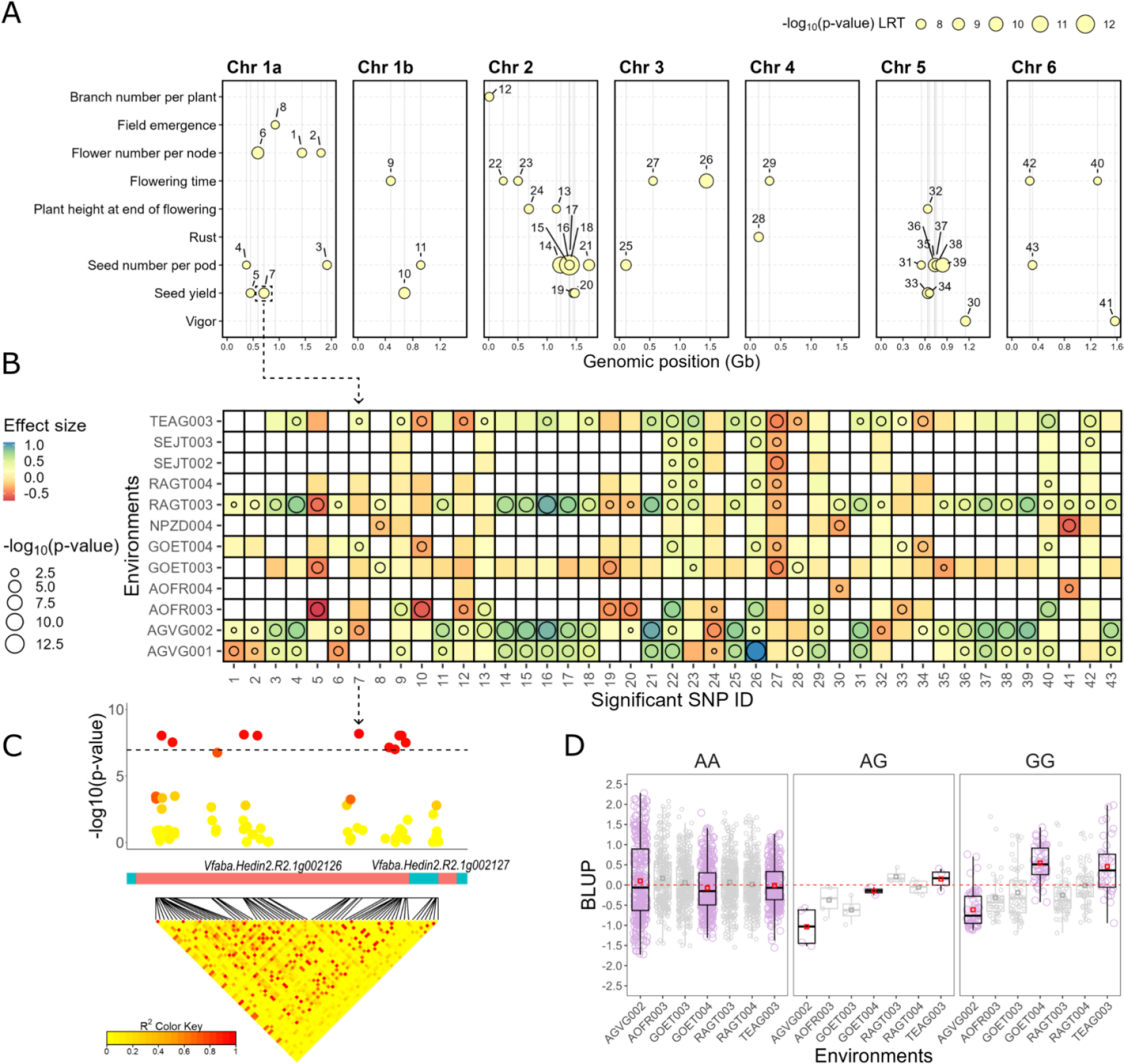
Genome-wide association analysis for genotype-by-environment interaction performed using linear mixed models. **A** significant SNPs are displayed for phenotypic traits along the genome coordinates. Numbers adjacent to SNPs correspond to detailed information provided in Additional file 2: Table S1. **B** For each marker detected as significant for the interaction effect shown in A, a Wald test was conducted for each coefficient of the QTLxE effects, and the results are presented in a heatmap. Circles within tiles indicate SNP and environment combinations where the *p*-value is below 0.01. White tiles represent missing trait x environment combinations, as not all traits were recorded in every environment. **C** Region plot showing the leading SNP (peak 7) and its LD with adjacent markers. **D** Boxplot of seed yield distribution per environment according to the genotype of SNP chr1a_707082783_A_G (peak 7). Distributions highlighted in purple show significant SNP effect (*P* ≤ 0.01) per environment category

The identification of QTL interaction with the environment is important to understand the relationship between genotype and phenotype as well as for targeted breeding of varieties for specific environments [10]. We started to map QTL expressing GxE by fitting an interaction model using plot-level data. Unlike a linear reaction norm model, which expects graded changes of the response variable along a continuous gradient of environmental quality, the interaction model is more flexible, allowing environment-specific effects to be scored. A total of 43 unique SNPs were detected with significant interaction effects (Fig. 3A, Additional file 2: Table S1). Most of these displayed conditional neutrality, an extreme case of differential sensitivity [11], where the SNP has a significant effect in some environments but no detectable signal in others. A more extreme manifestation of GxE happens when the QTL effect changes direction across environments, i.e., it displays an antagonistic pleiotropy effect. This type of effect was detected for 14 marker-trait associations, including signals 7, 20, 33, and 34 for seed yield, 30 and 41 for vigor, and 32 for plant height at the end of flowering (Fig. 3B). Signal 7 in Fig. 3 harbors a gene (*Vfaba.Hedin2.R2.1g002127*) that encodes a dehydration-responsive element-binding (DREB) transcription factor that confers resistance to cold among other stressors. Zhang et al. [21] identified the same gene located within the *FROST RESISTANCE-1 (FR-1*) locus using the ProFaba panel and validated its role in conferring freezing tolerance in faba bean, also showing that *FR-1* is the primary locus distinguishing winter from spring types. The heatmap of the region, with variant density augmented by whole-genome resequencing, reveals a tight cluster of accessions carrying the alternate allele, suggesting they share a similar haplotype structure, with the lead SNP as part of a haplotype block of limited size (Additional file 1: Fig. S11). Accessions containing the alternate allele yielded significantly less in Spain (AGVG002) as opposed to improved performance in Ireland (TEAG003) and Germany (GOET004), two of the coldest environments (Fig. 3D). Higher yield is generally reported for winter over spring type faba beans [22]. However, the QTL effect appears to be important even when winter types are sown in spring, with the direction of the effect depending on specific environmental conditions. A significant negative association is found when regressing the slopes of seed yield (synCov) on the SNP chr1a_707082783_A_G, indicating that the allele typically found in winter types confers an advantage in cooler and more humid climates, consistent with the *FR-1* locus characterization [21]. The alternate allele is associated with taller plants and longer flowering time, partially explaining the correlations of slope of seed yield and intercept of these traits (Additional file 1: Fig. S10). The introgression of the winter-associated allele into elite spring lines may enhance stability by triggering the CBF/DREB1 regulon under cold-induced stress conditions. Another marker displaying antagonistic pleiotropy effect is represented by hit 28, a significant peak detected for rust disease incidence. The SNP is located within the only gene in the candidate region and encodes a PECTIN METHYLESTERASE INHIBITOR, known for enhancing disease resistance [23] by inhibiting microbial pectin methylesterases. Additionally, whole genome resequencing revealed 10 missense variants within this gene, including high/impact ones (e.g., Phenylalanine to Serine) (Additional file 2: Table S9), making it a strong candidate. A putative FLOWERING LOCUS T gene was also discovered adjacent to a significant predictor (peak 32) with negative effect on plant height at the end of flowering in Spain but positive effect in Northern France and Ireland, thereby displaying antagonistic pleiotropy effect. A single missense variant early in the protein and several intronic ones were identified from resequencing data. This QTL harboring an ortholog of the FT gene has previously been reported explaining significant phenotypic variation for flowering time [12, 24]. All together, results indicate environment-specific effects for putative key genes, with sign changing effects for a subset, which strongly contribute to rank changes across environments.

### Association analysis of reaction norm coefficients

**Fig. 4:**
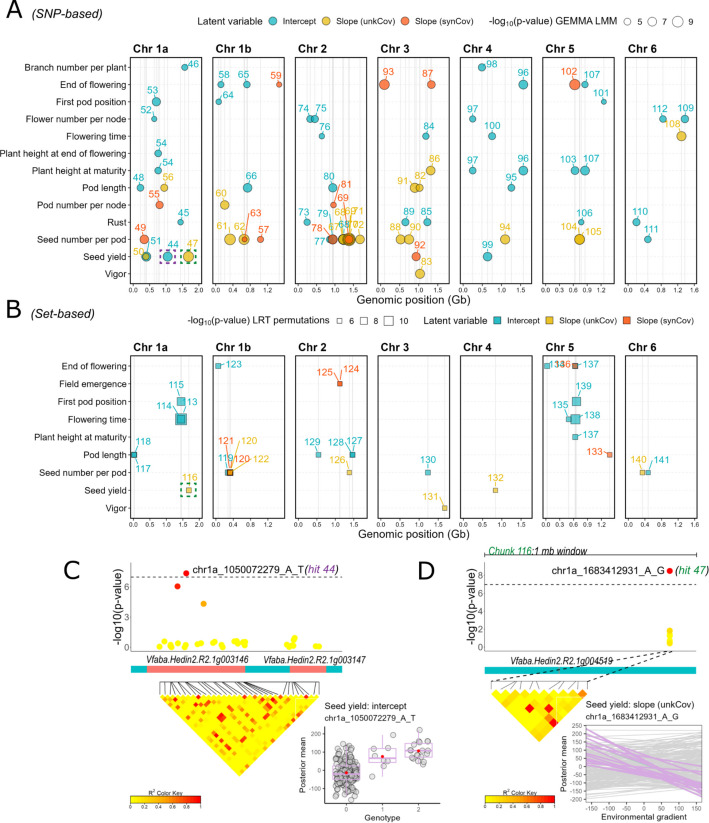
Genome-wide association studies of reaction norm coefficients. **A** Significant SNP markers from GWAS conducted on mean performance and stability defined by the latent variables intercept and slope (unkCov), respectively, of a reaction norm model with an unknown covariate as the environmental index. GWAS was also conducted on slopes (synCov) of a second reaction norm model where the environmental index is replaced by a synthetic covariate. Candidate SNPs were identified by multiple models with different assumptions of marker effect. *P*-values of the GEMMA-LMM model are depicted as -log10(*p*-value). Adjacent numbers link the significant SNPs with its detailed information presented in Additional file 2: Table S2. **B** Set-based association analysis conducted on the same traits and latent variables as in panel **A**. Set significance was detected via likelihood ratio test with 10 permutations used to compute *p*-values. Multiple testing was addressed via Bonferroni correction at 5%. Adjacent numbers link the significant set with its detailed information presented in Additional file 2: Table S3. **C** Region plot for the peak 44 highlighted in panel **A** with adjacent effect plot. Pairwise linkage disequilibrium (LD) for all variants found within ± 44.08 kb is shown as a heatmap, which covers 5.5 kb. **D** Region plot for the peak 47 (panel **A**) which is located within chunk 166 (panel **B**) with an adjacent plot showing the effect of SNP genotypes on reaction norms (faba bean lines containing the minor allele are highlighted). The LD heatmap is shown for variants within the 1 Mb region, spanning 83 bp

In addition to the GWAS performed using an interaction model, we conducted association analysis on reaction norm slopes and intercept (Additional file 3: Fig. S1). Marker-trait association analysis using single- and multi-locus models revealed a total of 75 significant associations with 69 unique SNP markers after clumping and filtering procedures (Fig. 4A). Significant marker-trait associations were detected for all recorded traits except field emergence. Additionally, associations spanned all three reaction norm-derived latent variables, with a greater number of significant hits detected for the intercept, from which a total of 59 candidate genes were identified either targeted by significant markers or located in their vicinity (Additional file 2: Table S2). Out of these associations, four SNP markers showed significant statistical effect for more than one of a set of three traits (hits 54, 96, 97, and 107). In general, SNPs detected for mean performance values were distinct from those associated with variation in stability. Exceptions occurred for seed yield with two colocalized markers (hits 50 and 51, Chr 1a) and seed number per pod, where the same marker was detected having significant effect for intercept and slope (unkCov) (hit 68, Chr 2).

We computed the variance explained of each marker-trait association by fitting all significant markers detected for a given trait plus principal components, to correct for population structure, in a random model [25]. Results revealed SNPs explaining from nearly zero to 50% of the observed variance of mean performance and stability across traits, with a median value of 14.52% (Additional file 2: Table S2). Three significant predictors were detected for the mean performance of seed yield (hits 44, 51, and 99; with variance explained of 16.42, 20.68, and 19.80%, respectively). A zoom into the locus where hit 44 was discovered reveals two candidate genes (Fig. 4C). Faba bean lines containing the minor, yield-promoting, allele belong to subpopulations sp2 and sp4, with origins in Central, Western, and Northern Europe (Additional file 2: Table S7). The phenotypic distribution for mean performance based on allele state for the additional associations identified is shown in Additional file 1: Fig. S7. The majority of significant markers were located within the coding region of genes, which is expected given that most of the SNPs originated from a targeting genotyping technology (SPET) with probes designed to largely target the exome.

Our GWAS analysis of reaction norm slopes revealed 22 marker-trait associations using the unkCov covariate method and 12 using the synthetic covariate (Fig. 4A; Additional file 1: Figs. S8 and S9), with 31 and 18 putative flanking genes, respectively. The most striking peak was detected for seed yield on chromosome 1a (hit 47) (Fig. 4A) and is also highlighted in Fig. 4D. The variance explained is 43.77% and the posterior probability of inclusion is equal to 27% (Additional file 2: Table S2). A single gene in the region (*Vfaba.Hedin2.R2.1g004519*) encodes a protein MAINTENANCE OF MERISTEMS (MAIN)-LIKE 2. A single marker-trait association (hit 92) was detected for slope of seed yield estimated using the synthetic covariate. This low-frequency marker accounts for nearly 50% of the variance in the ProFaba panel and is located within the only gene in the studied region, which encodes a TUBBY-like F-box protein 8. Variant consequence prediction based on whole genome resequencing data revealed several synonymous changes within the coding region, one missense variant (arginine-to-histidine substitution), and two variants in predicted splice regions (Additional file 2: Table S9). Faba bean lines carrying the minor allele belong primarily to sp1 and showed an increased yield above the population average as the environmental gradient shifted from cold and humid to warm and dry (Additional file 1: Fig. S9).

We also modeled reaction norm coefficients using a complementary approach to single-marker GWAS based on SNP sets as the testing units (Fig. 4B). The set-based association analysis can be more powerful by reducing the multiple-testing burden and by revealing associations due to the cumulative effect of multiple small-effect SNPs. We performed set-based analysis using both gene and 1 mb chunks as testing units. The analysis of mean performance revealed 13 significant chunks and 5 genes, three of which were located within chunk regions. Hit 114 refers to a gene encoding a homolog of FLOWERING LOCUS T (FT), a key florigen involved in photoperiod-mediated flowering regulation. This gene, *Vfaba.Hedin2.R2.1g004008*, is functionally annotated as a florigen component within the FT-FD floral activator complex. Its heritability was estimated at 0.45 (Additional file 2: Table S3). The smallest *p*-value (-log10) among the 19 predictors in the gene region detected for flowering time using GEMMA LMM was 5.054, which is below the defined genome-wide significance threshold of 6.964. This example demonstrates the benefit of complementing single-marker methods with set-based association analysis. The clustering pattern depicted in Additional file 1: Fig. S11 implies the presence of a haplotype block, which is shared among early flowering lines. Importantly, the gene harbors multiple missense and synonymous mutations, a splice-region variant, and a high density of intronic polymorphisms (Additional file 2: Table S9). These variants occur in both shared coding regions and the isoform-specific C-terminal extension of the encoded protein. The novel FT paralog on chromosome 1 shows strong sequence similarity (64% identity) with the known chromosome 5 *FT* gene mentioned earlier, expanding our understanding of the multi-locus genetic architecture controlling flowering time in *Vicia faba*. These dual signals likely reflect the characteristic duplication and expansion of the PEBP gene family commonly observed in temperate legumes [26]. Remote homology modeling using HHpred aligned these candidate sequences with the *Arabidopsis* FLOWERING LOCUS T crystal structure (PDB: 6IGG_A [27]) at 100% probability (Additional file 1: Fig. S12), a master florigen signal shown to act as the long-range mobile protein that triggers the transition to flowering [28]. For seed yield, results revealed two significant chunks, one of them (chunk 116) supporting the association highlighted in Fig. 4D.

We investigated the expression of 8 candidate genes located in single-gene QTL intervals. All pairwise trait-gene combinations were tested by linear regression (Additional file 1: Fig. S13), revealing that *Vfaba.Hedin2.R2.1g002122* expression was significantly associated with multiple traits. *Vfaba.Hedin2.R2.1g002122* was identified in GWAS analysis of first pod position. Increased relative expression of *Vfaba.Hedin2.R2.1g002122* was associated with reduced first pod position and plant height, supporting a role for this gene in regulation of overall height. *Vfaba.Hedin2.R2.1g002122* relative expression was also correlated, although to a lesser degree, with other developmental phenology and architecture traits, suggesting that the gene could exert pleiotropic effects (Additional file 1: Fig. S13). Based on Mercator functional annotation, the gene is a member of the ATP-binding cassette (ABC) transporter B family, specifically ABCB28 (Additional file 2: Table S2). Subcellular localization prediction (DeepLoc) suggests the protein is targeted to the plastid. While ABC transporters can be critical for plant growth and development, the specific substrate and biological function of *VfABCB28* remain to be determined.

### Genome-wide prediction of mean performance and stability

**Fig. 5:**
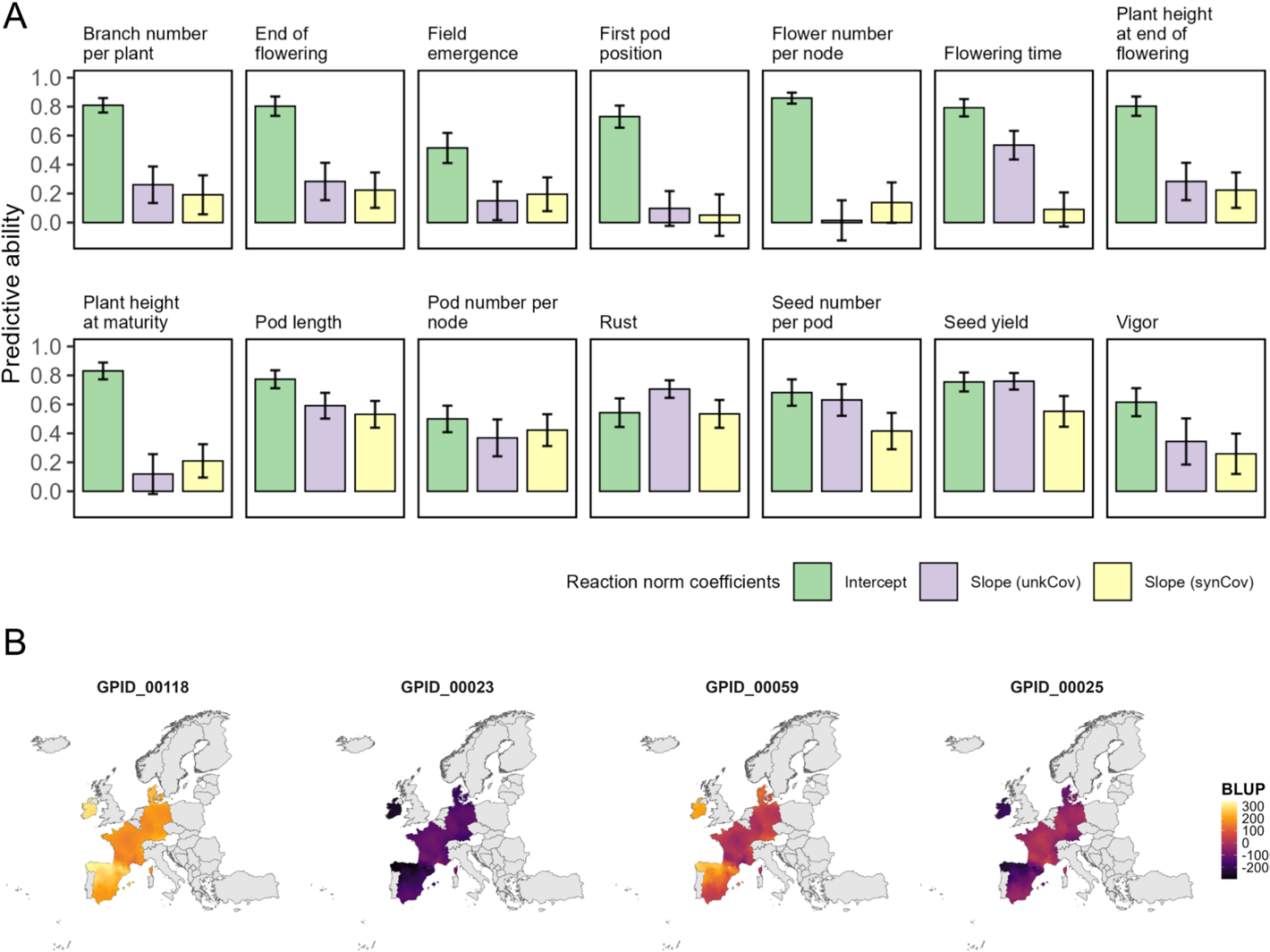
Prediction of mean performance and stability. **A** Predictive ability estimated for three reaction norm-based latent variables across 14 traits assessed on a faba bean diversity panel evaluated in multiple environments. Genomic prediction was performed using a GBLUP model, with genomic covariances estimated using 540 K SNP markers. Error bars refer to the standard deviation of correlations across 100 repetitions of a five-fold cross-validation scheme. **B** Thematic maps of predicted faba bean yield performance. Interpolated Best Linear Unbiased Prediction (BLUP) values of seed yield (g/m^2^) in 2020 is depicted for four faba bean accessions. The figures are arranged from left to right by highest intercept, lowest intercept, highest slope, and lowest slope values. Solutions of intercept and slope from a reaction norm model fitting a synthetic covariate were used to estimate mean performance across all random sampled coordinates across five European countries (see Fig. 2A). Interpolation was then performed using the inverse distance weighted (IDW) method with power equal to 2

We tested the extent to which mean performance and stability of untested lines could be predicted via a genomic BLUP model considering only additive (substitution) effects. We used a repeated 5-fold cross-validation approach and simple correlations between predicted and observed values as the metric of accuracy. Predictive ability for intercept values ranged from 0.50 for pod number per node to 0.86 for flower number per pod, and were generally higher than the estimates computed for slopes (Fig. 5A). Scale-corrected slope solutions from reaction norm models using either an unknown or a synthetic covariate were particularly predictable for traits showing substantial GxE (Additional file 1: Figs. S3 and S4), with the former having generally higher predictive abilities. Cross-validation showed a predictive ability of 0.75 for mean seed yield performance, 0.76 for the slope of a reaction norm using an unknown covariate, and 0.55 for the slopes obtained from the model using the synthetic covariate. The correlation between slope (unkCov) and slope (synCov) was |0.77| for seed yield (Additional file 1: Fig. S10). This finding suggests that the proposed method, which uses a principal component representing linear combinations of weather variables, performs similarly to the commonly used index based on environmental means. However, it offers the added advantage of enabling the prediction of line performance in untested environments. This task was evaluated via a leave-one-environment-out cross validation scheme, revealing predictive abilities in the same range as the standard prediction model accounting for GxE in plant breeding [29] (Additional file 3: Fig. S2).

From the reaction norm model using the synthetic covariate described in this study, predictions of line performance can be drawn across the multidimensional space on which true environments were projected. As an example, Fig. 5B presents thematic maps of the predicted seed yield for four accessions, chosen based on the extremes of their intercept and slope values, for the year 2020. Adaptation zones can be readily identified in such maps, improving the understanding of genotype-by-environment interaction for selection candidates. The adaptation patterns shown in Fig. 5B are constrained to the set of environmental factors with significant loadings on the first principal component (Fig. 2C). However, resolution can be maximized by extending the number of environmental variables and years used to construct the multidimensional space as well as by adding additional principal components as regressors on the modeling step.

## Discussion

Here, we investigated the genetic architecture of faba bean yield, yield stability, and a range of other agronomic traits, all assessed in a diversity panel tested across multiple environments. The resulting catalog of GWAS hits, along with the ProFaba phenotypic and genotypic data, provides a valuable resource for identifying genetic factors influencing key faba bean agronomic traits, supporting marker-assisted selection in breeding programs and facilitating quantitative genetic applications for crop improvement.

Although faba bean diversity panels have been used for mapping the main effects of QTLs [12, 13, 30, 31], QTL-by-environment interactions are seldom explored. Data from multi-environment trials are instead often aggregated into adjusted means or analyzed individually, primarily to identify stable signals. In contrast, our study reveals multiple QTLs associated with altered phenotypic response as a function of varying environments. Notably, we found that a major locus distinguishing spring and winter types [21] is also associated with environment-specific seed yield (Fig. 3C, D).

Reaction norm analysis of multi-environmental data can be fitted using random regression mixed models, a powerful approach to characterize plasticity and its variation [32]. Association analysis of reaction norm coefficients (intercept and slope) can reveal loci controlling mean performance and linear plasticity [8, 9, 19]. Fitting reaction norms with environmental means as covariates, we detected a major QTL on chromosome 1 explaining 43% of the variance of seed yield stability in the ProFaba panel (Fig. 4D). Stability is disrupted as accessions carrying the minor allele performed significantly below the population average with increasing environmental quality. However, the climate variables that influence the environmental means are unknown. This makes it challenging to predict performance outside of the observed environments, because an experimentally determined environmental mean, e.g. mean seed yield for a specific year/location, is required to determine the position on the x-axis, e.g. Figure 4D lower panel. This can be circumvented by replacing the environmental means with known covariates such as yield potential estimated using crop growth models with weather data as inputs [9], or environmental variables per se [33]. Post-processing of factor loadings from factor analytic mixed models can also be performed to identify associated environmental variables [34, 35]. However, the multiple modeling steps involved require data from a relatively large number of environments. A simpler approach is to fit a mixed model with a relationship matrix based on environmental variables [29], but this also has limitations [36, 37], including restrictive assumptions for variances and covariances of regression terms [38].

Here we developed a method to include environmental information in the analysis of genotype-by-environment interaction, which allows predicting genotype performance in unseen environments in a straightforward manner without relying on multiple modeling steps. With a single synthetic covariate, the reaction norm model was able to approximate the GxE variance explained using environmental means for some traits and explain more GxE variance for others, uncovering candidate genes that were undetected with the traditional approach. The proposed approach addresses some of the constraints of the current methods: The dimensionality reduction step removes the need for variable selection; the computational burden is limited as only one or a few synthetic covariates are sufficient to explain most of the environmental variance; and analysis can be performed with as few as two environments, provided that they represent the extremes of the target population of environments [7].

## Conclusions

Our work advances the understanding of the genetic basis of faba bean traits and trait stability. It also provides a general framework for incorporating complex climate data into reaction norm models, substituting environmental means to allow prediction of genotype performance in unseen environments. This now allows initiation of breeding for improvement of yield stability for faba bean and other crops where yield is challenged by environmental variation, including projected future climate changes.

## Methods

### Plant material and phenotype acquisition

This study utilizes the ProFaba diversity panel, comprising a collection of faba bean inbred lines from diverse origins, which were phenotyped across multiple environments in Europe. We focused on 222 accessions from the original panel, excluding lines with phenotypes recorded in only one environment. Field trials were conducted across seven locations over one or two crop seasons, resulting in 12 distinct environments defined by combinations of location and year, spanning 18.4 degrees in latitude and 16.8 degrees in longitude (Additional file 2: Table S5). In each environment, accessions were assigned to two complete blocks. Trials were established in the spring at all locations, except in Spain, where sowing occurred in the fall. Information on accession names and their countries of origin is provided in Additional file 2: Table S7. The diversity panel was phenotyped for several traits, of which 14 were selected based on a normal distribution of records and measurements taken in three or more locations. The list of traits and the scoring procedure can be found in Additional file 2: Table S6.

### Genotyping and data processing

The genome assembly of the ‘Hedin/2’ faba bean [14] allowed the targeting of predicted genes with a 90 K probes for high-throughput Single Primer Enrichment Technology (SPET) genotyping. Shortly, libraries were prepared using the Allegro Targeted Genotyping protocol (NuGEN Technologies, San Carlos, CA) and sequenced in an Illumina NovaSeq 6000 (Illumina, San Carlos, CA) with a 2 × 150 PE configuration. Additionally, genotyping-by-sequencing (GBS) was also performed based on an optimized procedure for faba bean [13]. Short reads from both the GBS and SPET platforms were merged and re-mapped to the version 2 of the ‘Hedin/2’ reference genome [21] for joint SNP calling (Additional file 2: Table S11). Stringent filtering criteria was applied to minimize false-positive heterozygous calls: only heterozygous sites with a genotype depth (DV) > 2, genotype quality (GQ) > 40, total site depth (DP) > 80, and a DV/DP ratio between 0.3 and 0.7 were retained. The final VCF contained nearly 90 million variants, which were filtered based on the hard-quality filters: overall quality (QUAL) score lower than 20 and mean mapping quality (MQ) lower than 40. Additionally, multiallelic and monomorphic sites as well as indels and variants in close proximity to indels (within 5 bp) were discarded. Finally, variants were filtered by missingness (20% or less of tolerance), heterozygosity (10% or less), and minor allele frequency (MAF) of 0.03. Variant Call Format (VCF) handling and all filtering steps were performed using BCFtools (v1.17) [39]. The remaining missing genotypes were imputed using the population-based imputation method implemented in Beagle 5.4 [40] with the arguments tuned via grid search after masking 5% of the genotype table, resulting in the value of 100 for effective population size (ne), 10 for window size, 2 for the overlap parameter, and 50 for the number of interactions. After imputation, the genotype table was filtered by MAF of 0.05, resulting in a set of 540 K of high-quality SNPs. A reduced set of 220 K of nearly uncorrelated SNPs was obtained after a linkage disequilibrium-driven clumping procedure executed using Plink (v1.9) [41]. Clumping was performed using the parameters –clump-p1 1, –clump-p2 1, –clump-r2 0.2, –clump-kb 250, and for the --clump argument, values of 1-MAF were supplied. This reduced set of markers was then used to infer genomic relationships among entries and derive principal components for downstream analyses. Finally, it is important to note that chromosome 1 was split into two (Chr 1a and Chr 1b) given its outlier size.

10-fold resequencing data of 33 lines of the NORFAB diverse panel [14] overlapping with the ProFaba panel (Additional file 2: Table S10) was used for variant validation, investigation of genomic regions harboring significant marker-trait associations, and prediction of variant consequences for candidate genes. Similar filters were applied to the resequencing data (missingness ≤ 25%, heterozygosity ≤ 10%, MQ ≥ 30, and MAF ≥ 0.05), yielding 135.7 million variants.

### Population structure and linkage disequilibrium

Population structure analysis of the diversity panel was performed using ADMIXTURE (v1.3.0) [15] using a subset of 102 K SNP variants, which was obtained after LD-based pruning with Plink (v1.9) [41] argument --indep-pairwise 50 10 0.1. The appropriate number of clusters (K) was determined by the smallest error produced by a 5-fold cross-validation on K varying from 2 to 10. The same subset of variants was used for principal component analysis with Plink v1.9. The first two eigenvectors were retrieved to create a scatter plot with inbred lines colored by subpopulation as identified by the admixture analysis. Linkage disequilibrium (LD) estimation was also performed with Plink (v1.9) [41] with arguments --ld-window 999999, --ld-window-kb 1000, --ld-window-r2 0, and --r2 aiming to calculate LD as squared correlation in the range of 0 to 1 within 1mb windows. The resulting LD data was sorted according to SNP distance and binned into groups of 100 data points, average LD ($\overline{R}^{2}$) per bin was computed, and a smoothing (LOESS) curve was fitted to the $\overline{R}^{2}$ as a function of the distance. The distance to the half-maximum value of the fitted curve was reported as the LD decay. Additionally, genome-wide nucleotide diversity (π) was calculated using TASSEL (v5.0) [42]. Finally, we calculate the realized genomic relationship matrix following VanRaden's method 1 [43] as $\mathbf{G}={\mathbf{ZZ}'}/2\sum {p}_{i} \left(1-{p}_{i}\right)$, where the numerator represent the cross-product of centered genotype scores and the denominator scales **G** to be analogous to the numerator relationship matrix **A**, computed with pedigree information, and ${p}_{i}$ is the reference allele frequency for SNP $i$. We then plotted **G** as a network using the R package igraph (v2.0.3) [44].

### Single stage genome-wide analysis of G × E

Data quality checks were performed to verify the adherence to the assumptions of the models implemented thereafter. Skewness were addressed via exponential transformation for rust score, raising each data point to a constant exponent equal to 2. Extreme outliers were detected by the Tukey’s fence method with K equal to 3 and removed from downstream analyses. Pre-processed phenotypic records were then modeled aiming to identify SNPs displaying interaction with the environmental component. This process was performed in a single step with the full data set using linear mixed models accounting for the genotype main effects and G × E (GGE) [10] using the R package gaston (v1.6) [45]. Initially, the following null model was fitted to the data:

LMM 1 $$\begin{array}{cc}\mathbf{y}=\mathbf{X}\beta +\mathbf{S}\mathbf{s}+{\mathbf{u}}_{G}+{\mathbf{u}}_{GE}+{\mathbf{u}}_{R}+{\boldsymbol{e}} \end{array}$$

where, **y** is a vector of phenotypic records, which was standardized within environment to have mean zero and variance one, β is the fixed effect of environment and complete replicate nested within environment, with an associated design matrix **X**; **S** contains the first three eigenvectors from the eigen decomposition of the **G** matrix and $\mathbf{s}$ is a vector with the respective fixed regression effects; **u**_*G*_, **u**_*GE*_, and **u**_*R*_ are the random effects of genotype, genotype-by- environment interaction, and row position of field plots, respectively, and are assumed as follows:

$${\mathbf{u}}_{G}\sim MVN(\mathbf{0}, \left[{\mathbf{Z}}_{\mathbf{G}}{\mathbf{G}\mathbf{Z}}_{\mathbf{G}}^{\mathbf{^{\prime}}}\right]{\sigma}_{G}^{2})$$

$${\mathbf{u}}_{GE}\sim MVN(\mathbf{0},\left[{\mathbf{Z}}_{\mathbf{G}}{\mathbf{G}\mathbf{Z}}_{\mathbf{G}}^{\mathbf{^{\prime}}}\right]\circ \left[{\mathbf{Z}}_{\mathbf{E}}{\mathbf{Z}}_{\mathbf{E}}^{\mathbf{^{\prime}}}\right]{\sigma}_{GE}^{2})$$

$${\mathbf{u}}_{R}\sim MVN\left(\mathbf{0},\left[{\mathbf{Z}}_{\mathbf{R}}{\mathbf{Z}}_{\mathbf{R}}^{\mathbf{^{\prime}}}\right]{\sigma}_{R}^{2}\right)$$

where $\circ$ indicates the Hadamard product and **Z**_**G**_, **Z**_**E**_, and **Z**_**R**_ are the incidence matrices of the random effects of genotype, environment, and row position, respectively. Narrow-sense heritability *h*^2^ was computed from variance components estimated in LMM 1 according to the following expression:

$${h}^{2}={\sigma}_{G}^{2}/\left({\sigma}_{G}^{2}+{\sigma}_{R}^{2}+\frac{{\sigma}_{GE}^{2}}{e}+\frac{{\sigma}_{e}^{2}}{e\cdot r}\right)$$

where, e is the number of environments and *r* is the harmonic mean of the number of replicates.

An SNP additive main effect ($\alpha$) is estimated by fitting the following LMM to each marker:

LMM 2 $$\begin{array}{cc}\mathbf{y}=\mathbf{X}\beta +\mathbf{S}\mathbf{s}+\mathbf{x}\upalpha +{\mathbf{u}}_{G}+{\mathbf{u}}_{GE}+{\mathbf{u}}_{R}+\mathbf{e}\end{array}$$

where $\mathbf{x}$ is a nx1 vector of maker genotypes coded as 0, 1, or 2. Finally, an SNP effect for each environment is obtained via model LMM 3, which takes the form:

LMM 3 $$\begin{array}{cc}\mathbf{y}=\mathbf{X}\beta +\mathbf{S}\mathbf{s}+\sum\limits_{l}^{L}\left\{\left({{\boldsymbol{\pi}}}_{l} \circ \mathbf{x}\right){\zeta}_{l}\right\}+{\mathbf{u}}_{G}+{\mathbf{u}}_{GE}+{\mathbf{u}}_{R}+\mathbf{e} \end{array}$$

where, ${\zeta}_{l}$ is the SNP effect in the *l-th* environment. Log-likelihood (LL) ratio tests (LRT) for models LMM 1 and 2 tests a marker-trait association common to all environments and LMM 2 and 3 tests the existence of maker G × E [10]. A Wald test for each coefficient of marker G × E was calculated. We then retained markers showing significant G × E effect (0.05/*M*_*eff*_) via LRT that also satisfied the condition of having significant effects (*p* < 0.01) via Wald test in at least three environments. The term *M*_*eff*_ refers to the effective number of independent tests computed via SimpleM method [46] with a window size equals to 20. Genomic inflation factor ($\lambda$) was monitored across all GWAS runs. Values of $\lambda$ ranged from 0.90 to 1.04 for the test statistic of the interaction effect, with an average of 0.97 across traits.

### Reaction norm with unknown environmental covariate

A reaction norm model was fitted to the data following an approach where the environmental covariate representing environmental quality is treated as an unknown variable and inferred simultaneously with other model parameters in a Bayesian framework [17]. In this text, we refer to this covariate as "unkCov," a shorthand for "unknown covariate". The univariate linear reaction norm model applied to the data is represented in the following matrix format:

RNM 1 $$\begin{array}{cc}\mathbf{y}=\mathbf{1}\mu +\mathbf{W}\mathbf{h}+{\mathbf{Z}}_{\mathbf{0}}{\mathbf{a}}_{\mathbf{0}}+{\mathbf{Z}}_{\mathbf{1}}{\mathbf{a}}_{\mathbf{1}}+\mathbf{Q}\mathbf{r}+\mathbf{e}\end{array}$$

where **y** is the vector of observations, **1** is a vector of 1’s and *µ* is the overall mean, **h** represents the random effect of environments (i.e., year-location-replicate combinations) following $N\left(0,\mathbf{I}{\sigma}_{h}^{2}\right)$, where **I** is the identity matrix, and with associated incidence matrix **W**, **Z**_0_, and **Z**_1_ connecting phenotypic observations to the random effects of intercept (**a**_0_) and slope (**a**_1_), which assume the following (co)variance structure:

$$\begin{aligned}\begin{bmatrix} \mathbf{a}_0 & \mathbf{a}_1 \end{bmatrix}^T = (0, \mathbf{I} \otimes \mathbf{V}), \mathbf{V} = \begin{bmatrix} \sigma_{a_0}^2 & \sigma_{a_0 a_1} \\ \sigma_{a_0 a_1} & \sigma_{a_1}^2 \end{bmatrix}\end{aligned}$$

Each row of the design matrix **Z**_**1**_ contain one non-zero element equal to the effect of environment where the measurement was recorded, which is extracted from $\mathbf{h}_{i-1}$, i.e., the environment effect from the previous sample of the Gibbs algorithm. $\mathbf{Q}$ is the design matrix for the random effect $\mathbf{r}$ assumed $N (0, \mathbf{I}\sigma^2_{r(h)})$ which has a diagonal structure with a variance component per environment and aim to capture the spatial variation of the row dimension, and $\mathbf{e}$ is the random residual vector following $N(0, \mathbf{R}\sigma_e^2)$, where $\mathbf{R}$ is a diagonal matrix allowing for heterogeneous residual variances over trials. The genetic (co)variance matrix was computed within the limit range of environments as $\boldsymbol{\Phi} \mathbf{V} \boldsymbol{\Phi}^{\boldsymbol{\prime}}$, where $\mathbf{V}$ is a 2 × 2 (co)variance matrix for intercept and slope and $\boldsymbol{\Phi}$ is an nx2 matrix containing a column of 1 s and the environmental covariate and n is the number of environments. The model was fitted using the RJMC module of DMU (v6.5.6) software [47] with 100,000 cycles of the Gibbs sampler. After discarding the first 20,000 samples as the burn-in period, every 20th sample was retained for the calculation of posterior means and standard errors of variance components. Convergence was verified by visual inspection of trace and Geweke’s convergence diagnostic plots using the R package coda [48].

### Reaction norm with a weather-derived covariate

A second reaction norm model was fitted to the data. Unlike RNM 1, the non-zero elements of the incidence matrix $\mathbf{Z}_\mathbf{1}$ now correspond to values of an external covariate, referred to as "synCov" (short for "synthetic covariate"), which is constructed as a linear combination of environmental variables. The procedure to obtain the weather-derived or synthetic covariate involved sampling 200 coordinates per country where field trials were carried out. Next, environmental variables were retrieved for each one of these points and consisted of daily averages of mean temperature (T2M), mean maximum temperature (T2M_MAX), root zone soil wetness (GWETROOT), precipitation corrected (PRECTOTCORR), evapotranspiration energy flux (EVPTRNS), wind speed (WS2M), relative humidity (RH2M), photosynthetically active radiation (ALLSKY_SFC_PAR_TOT), and clear sky day (CLRSKY_DAYS) for the period within the start and end date of the field trial for each crop season and country. Weather data was fetched from the NASA POWER API using the R package nasapower (v4.2.1) [49]. Environmental variables were summarized for the entire crop season by computing averages. A filtering step was applied where only distinct values of the environmental covariates were allowed, therefore removing duplicate entries due to geographical proximity. The final data set consisted of 299 random points and 9 variables, which was used for principal component analysis. Component significance was investigated via permutation test using the R package syndRomics (v0.1.0) [50]. Permutation was also used to assess the contribution (loadings) of each variable and its direction on the principal component in order to define a component identity. Next, weather data from the actual field trial locations was used to project these locations onto the PCA space by multiplying the new data with the rotation matrix. Finally, the synthetic environmental covariate was extracted and refers to the scores of the first principal component for the tested environments. Therefore, the engineering of the synthetic covariate remained an unsupervised process and only dependent on the weather similarities among environments within the time span of field testing. A Bayesian reaction norm model that regress entries on an index defined by the synthetic covariate was then fitted to the data. The model in matrix notation is as follows:

RNM 2 $$\mathbf{y} = \mathbf{1}\mu + \mathbf{X}\beta + \mathbf{Z}_\mathbf{0} \mathbf{a}_\mathbf{0} + \mathbf{Z}_\mathbf{1} \mathbf{a}_\mathbf{1} + \mathbf{Qr} + \mathbf{e}$$

where, $\beta$ is a vector of fixed effects of year-location-replicate classes with associated incidence matrix $\mathbf{X}$. The remaining terms and assumptions are as defined for model RNM 1 as well as the implementation and convergence diagnostics.

### Genome-wide association analysis of reaction norm model coefficients

Solutions of intercept and slope produced by model RNM 1 and slopes of model RNM 2 were utilized as response variables in genome-wide association studies. Prior to GWAS, solutions of slope of both models were subjected to a correction in order to remove the component of GxE that is due to differences in genetic variance along the environmental index, named scale-type GxE. The correction was performed by applying a genetic regression [20], which takes the following form:

$$\mathbf{a}^*_{\textit{slope}} = \mathbf{a}_{\textit{slope}} - \frac{\sigma_{a_{\textit{int}}a_{\textit{slope}}}}{\sigma^2_{a_{\textit{int}}}}\mathbf{a}_{\textit{int}}$$

where $\sigma^2_{a_{int}}$ is the intercept variance and $\sigma_{a_{\mathit{int}} a_{\mathit{slope}}}$ is the covariance between intercept and slope. Therefore, we focus on the rank-type component of plasticity, which is due to the imperfect correlation of genotypic values across environments. Association analysis of mean performance of inbred lines across environments was exclusively conducted for intercept values of model RNM1, as they show nearly-unit correlation with solutions of intercept from model RNM2. Collectively, coefficients intercept and slope are treated as latent variables throughout the text. Univariate single-marker analysis was performed using Genome-wide Efficient Mixed Model Analysis implemented in GEMMA (v0.98.5) [51] following a linear model of the form: $\mathbf{y} = \mathbf{W}\boldsymbol{\alpha} + \mathbf{x}\boldsymbol{\beta} + \mathbf{u} + \mathbf{e}$, where $\mathbf{y}$ is the response vector; $\boldsymbol{\alpha}$ is a vector of fixed effects (a constant plus the first three principal components) with associated design matrix $\mathbf{W}$; $\mathbf{x}$ is a vector of the SNP marker being tested and $\beta$ is its estimated effect size; $\mathbf{u}$ is the random polygenic term assumed $\boldsymbol{u} \sim \mathrm{MVN}(0, \mathbf{K}\sigma_g^2)$, where $\mathbf{K}$ is the centered relatedness matrix computed as $\mathbf{K} = \mathbf{X}\mathbf{X}^{\mathrm{\prime}}/p$, where $\mathbf{X}$ is the matrix of genotypes and $p$ is the number of clumped markers. GEMMA was also used to fit the Bayesian Sparse Linear Mixed Model (BSLMM), a multilocus model that takes the form $\mathbf{y} = \mathbf{1}_n \mu + \mathbf{X}\boldsymbol{\beta} + \mathbf{u} + \mathbf{e}$, where $\mathbf{1}_n$ is a vector of 1 s, $\mu$ is the overall mean, $\mathbf{X}$ is a matrix of genetic markers with the associated vector of sparse marker effects $\beta$, where SNP effect is sampled from a mixture of two distributions $\beta_{i} \sim \pi \mathrm{N}(0, \sigma_{a}^{2} + \sigma_b^2) + (1-\pi) \mathrm{N}(0, \sigma_b^2)$, with probability $\pi$ of $\beta_i$ being large and probability $1-\pi$ of $\beta_i$ being small, $\sigma_b^2$ and $\sigma_{a}^{2}$ are the associated variances due to small and large effect sizes, respectively. BSLMM was run for 3 million MCMC interactions, with 0.5 million as burn-in period, and 25 for the recording pace argument. Convergence diagnostics were performed as specified before for the reaction norm models. Another multiple loci approach implemented was BLINK [52], which stands for Bayesian-information and linkage-disequilibrium iteratively nested keyway. Here, two fixed effect models are fitted iteratively, the first tests all markers, one at a time, while including additional markers as cofactors, named pseudo quantitative trait nucleotides (QTNs). The second model aims to optimize the selection of pseudo QTNs to be included in the first model aiming to control false positives and reduce false negatives. The GWAS using BLINK was conducted by GAPIT (v3.5) [25].

In addition to single-marker regressions, two variations of set-based analysis were performed on latent variables using the software LDAK (v5.2) [53] in a leave-one-chromosome-out (LOCO) setting. Firstly, we performed gene-based analysis where sets were defined by genomic coordinates of annotated genes, with 2 kb buffer on each end (argument --gene-buffer 2000) and a minimum of 9 predictors per gene (argument --min-weight 9). In total, 18,618 genes were tested with a median of 21 predictors each. Secondly, the genome was scanned by overlapping windows of 1 mb in length. In total, 17,506 windows with a median of 45 predictors were tested for association.

All models except BSLMM were fitted using the first three eigenvectors as covariates to address population stratification. For those models, significance was declared when *p*-values exceed a threshold defined by $-{log10}(0.05/M_{eff})$. The significance threshold was then set to 6.964. For gene- and set-based analysis, a Bonferroni threshold at 5% was used. The final set of candidate variants consisted of SNPs with BLINK *p*-values surpassing the genome-wide significance threshold, along with GEMMA-LMM *p*-values lower than 0.001, or with GEMMA-LMM *p*-values exceeding the genome-wide threshold. In both cases, only variants with a non-zero posterior probability of inclusion (PIP), as derived from the BSLMM model, were retained. Furthermore, clumping was performed for 40 kb windows, eliminating variants with a squared correlation exceeding 0.1 with the SNP displaying the lowest *p*-value. For the set-based analyses, clumping was performed with window size set to the whole chromosome. While set-based analysis returns an estimate of set heritability, variance explained by single markers was calculated via a random model similar to the GAPIT3 implementation [25].

### Candidate identification and expression assay

We searched for putative protein-coding genes flanking all discovered loci, within a search window of ± 44.08 kb. For seed yield, region plots were constructed showing the leading SNP and its linkage disequilibrium (LD) with adjacent markers. Additionally, pairwise LD between all markers in the region was graphically displayed as a heatmap using the R package LDheatmap (v0.99.6) [54]. Candidate gene function annotation was performed by loading predicted protein-coding sequences into Mercator4 [55] with the option to utilize Prot-scriber and Swissprot annotations. Multi-tissue gene expression data from a previously published study [56] was appended to candidate genes to provide insight into tissue-specific expression patterns. Finally, haplotype-aware variant consequences were called using BCFtools [57] csq command.

We searched for QTL regions harboring, preferably, a single gene for further validation via RT-qPCR. The selected genes along with the their association IDs (Additional file 2: Tables S1 to S3) are as follow: *Vfaba.Hedin2.R2.1g003146* (44), *Vfaba.Hedin2.R2.1g003147* (44), *Vfaba.Hedin2.R2.4g024979* (99), *Vfaba.Hedin2.R2.1g002282* (54), *Vfaba.Hedin2.R2.1g002122* (53), *Vfaba.Hedin2.R2.1g004001* (45), *Vfaba.Hedin2.R2.1g004008* (113), *Vfaba.Hedin2.R2.5g030166* (32, 103). For each gene, we identified lines with extreme phenotypes based on the allele of the significant GWAS variant, selecting approximately 12 lines carrying the reference allele and 12 carrying the alternate allele. Altogether, 60 lines were required to evaluate all eight genes. Seeds from the selected lines were germinated on plates containing two layers of germination paper, with approximately 10 mL of sterile ddH₂O added to each plate. The plates were incubated at 25 °C. After two days, germinated seeds were transferred to pots filled with a mixture of soil and LECA (light expanded clay aggregate), with four to five seedlings planted per pot. Ten days after germination, second leaves were harvested for RNA extraction. RNA was extracted using a commercial kit following the manufacturer’s instructions. To eliminate DNA contamination, DNase I (Cat. 2943769) was applied, and reverse transcription was performed using Maxima H Minus Reverse Transcriptase (Cat. EP0753). Quantitative PCR was carried out using LightCycler 480 SYBR Green I Master (Cat. 04887352001) according to the manufacturer’s protocol. Each sample was run in triplicate to ensure reproducibility. Relative expression levels of the candidate genes were calculated using the 2^(− ΔΔCt) method, with *Vfaba.Hedin2.R2.3g020816* used as the reference gene for normalization. Finally, phenotypic traits were regressed on relative gene expression levels, with multiple testing accounted for using false discovery rate (FDR) correction. Protein subcellular localization was investigated using DeepLoc 2.0 [58]. Remote structural homology was verified using the HHpred server via profile HMM comparisons against the Protein Data Bank [59]. Sequence and structural alignments were formatted and visualized using the ESPript 3.0 web server [60].

### Evaluation of prediction performance

A genomic prediction study was performed on the on reaction norm-derived latent variables intercept and slope of model RNM1, which uses an unknown covariate in the estimation process, and slopes of model RNM2, in which a synthetic covariate is explicitly defined. We used a genomic BLUP (GBLUP) model of the form: $\mathbf{y} = \mathbf{1}\mu + \mathbf{Z}\mathbf{u} + \mathbf{e}$, where $\mathbf{y}$ is the response vector (latent variable), $\mu$ is a constant with associated vector $\mathbf{1}$ containing 1 s, $\mathbf{u}$ is the random vector of genomic estimated breeding values with associated design matrix $\mathbf{Z}$ and assumed $\mathbf{u} \sim N(\mathbf{0}, \mathbf{G}\sigma_g^2)$, where $\mathbf{G}$ is the realized genomic relationship matrix as defined before, and $\mathbf{e}$ is the random vector of residues assumed $\mathbf{e} \sim N(\mathbf{0}, \mathbf{I}\sigma_e^2)$. We implemented a 5-fold cross-validation approach repeated 100 times to assess the predictive ability of the model, measured as the correlation between the observed and predicted BLUPs of intercept and slopes. The model was fitted using the mixed.solve function of the R package rrBLUP (v4.6.3) [61].

In a second prediction study, the proposed method based on random regression on a synthetic covariate was compared to the well-known multiplicative reaction norm model proposed by Jarquin et al. (2014) [29]. For this end, we expanded model RNM2 to accommodate random regressions on the first two principal components, which were highly significant and accounted for 65% of the variance (Fig. 2B). Therefore, the model RNM2 includes an additional term for the random slopes of the second principal component. For the multiplicative reaction norm model, the model terms $\mathbf{Z}_\mathbf{1} \mathbf{a}_\mathbf{1} + \mathbf{Z}_\mathbf{2} \mathbf{a}_\mathbf{2}$ were replaced by a random genotype-by-environment interaction term ($\mathbf{Z}_{\mathbf{ge}} \mathbf{a}_{\mathbf{ge}}$), assumed to follow a multivariate normal distribution $\mathbf{a}_{\mathbf{ge}} \sim N(\mathbf{0}, \boldsymbol{\Omega} \otimes \mathbf{G})$, where $\boldsymbol{\Omega}$ is the environmental relationship matrix computed as $\boldsymbol{\Omega} = \mathbf{WW}^{\boldsymbol{\prime}}/q$, where $\mathbf{W}$ is the matrix of centered and scaled environmental variables and $q$ is the number of environmental variables, and $\mathbf{G}$ is the genomic relationship matrix as defined before. Models were compared on their ability to predict line performance in new environments following a leave-one-environment-out cross-validation scheme. For each left out environment, the vector of genomic estimated breeding values of all lines was correlated with adjusted means, obtained by fitting a linear mixed model considering lines as fixed effect and replicate-row classes as random. Models were fitted using DMU (v6.5.6) [47], with module DMUAI for variance component estimation and module DMU4 for cross-validation.

## Supplementary Information

Additional file 1: Supplementary Figures. Contains Figs. S1–S13. Fig. S1. Variant density. Fig. S2. Density distributions of trait values across environments. Fig. S3. Genetic correlations from a Bayesian reaction norm model with an unknown covariate. Fig. S4. Genetic correlations from a Bayesian reaction norm model with a synthetic covariate. Fig. S5. Genetic variance along the environmental gradient from a Bayesian reaction norm model with an unknown covariate. Fig. S6. Genetic variance along the environmental gradient from a Bayesian reaction norm model with a synthetic covariate. Fig. S7. Effect plots of significant marker-trait associations for trait mean values. Fig. S8. Effect plots of significant marker-trait associations for slopes of a reaction norm model with an unknown covariate. Fig. S9. Effect plots of significant marker-trait associations for slopes of a reaction norm model with a synthetic covariate. Fig. S10. Correlation between posterior means of trait mean and stability. Fig. S11. Haplotype view of genomic regions harboring significant associations. Fig. S12. Protein homology detection via HHpred. Fig. S13. Regression analysis of trait values on candidate gene expression

Additional file 2: Supplementary Tables. Contains Tables S1–S11. Table S1. Association results for the QTL × E single-stage analysis. Table S2. Multi-model GWAS for mean performance and stability. Table S3. Set-based association analysis of mean performance and stability. Table S4. List of significant SNPs. Table S5. Description of testing environments. Table S6. List of traits. Table S7. Description of the ProFaba panel. Table S8. Meteorological parameters. Table S9. Variant consequences. Table S10. List of resequenced lines. Table S11: Mapping and quality statistics for sequencing reads

Additional file 3: Extended data. Contains Table S1 and Figs. S1 and S2. Table S1. Posterior estimates for genetic parameters. Fig. S1. Reaction norms. Fig. S2. Model comparison via cross-validation

## Data Availability

The complete collection of ProFaba traits and field trials along with the curated dataset of phenotypic data produced during this study as well as the SNP marker data is openly available in a Zenodo repository [16]. Resequencing reads are available in ENA under accession PRJEB94134 [62] and SPET reads under accession PRJEB85172 [63]. All software packages used in this study are specified in the Methods section and are publicly available. Data manipulation and visualization were performed using R (v4.4.0) (https://www.r-project.org).
