# Supplementary material for: Dissecting the genetic basis of yield stability in faba bean by multi-environment analysis
Source: Genome Biol. 2026 Jul 20;27:231. doi: 10.1186/s13059-026-04181-0 (PMC13383516; doi:10.1186/s13059-026-04181-0)
Supplement: Supplementary file 1 — Additional file 1: Supplementary Figures. Contains Figs. S1–S13. Fig. S1. Variant density. Fig. S2. Density distributions of trait values across environments. Fig. S3. Genetic correlations from a Bayesian reaction norm model with an unknown covariate. Fig. S4. Genetic correlations from a Bayesian reaction norm model with a synthetic covariate. Fig. S5. Genetic variance along the environmental gradient from a Bayesian reaction norm model with an unknown covariate. Fig. S6. Genetic variance along the environmental gradient from a Bayesian reaction norm model with a synthetic covariate. Fig. S7. Effect plots of significant marker-trait associations for trait mean values. Fig. S8. Effect plots of significant marker-trait associations for slopes of a reaction norm model with an unknown covariate. Fig. S9. Effect plots of significant marker-trait associations for slopes of a reaction norm model with a synthetic covariate. Fig. S10. Correlation between posterior means of trait mean and stability. Fig. S11. Haplotype view of genomic regions harboring significant associations. Fig. S12. Protein homology detection via HHpred. Fig. S13. Regression analysis of trait values on candidate gene expression [file 13059_2026_4181_MOESM1_ESM.docx]

**Supplementary Figures**

**
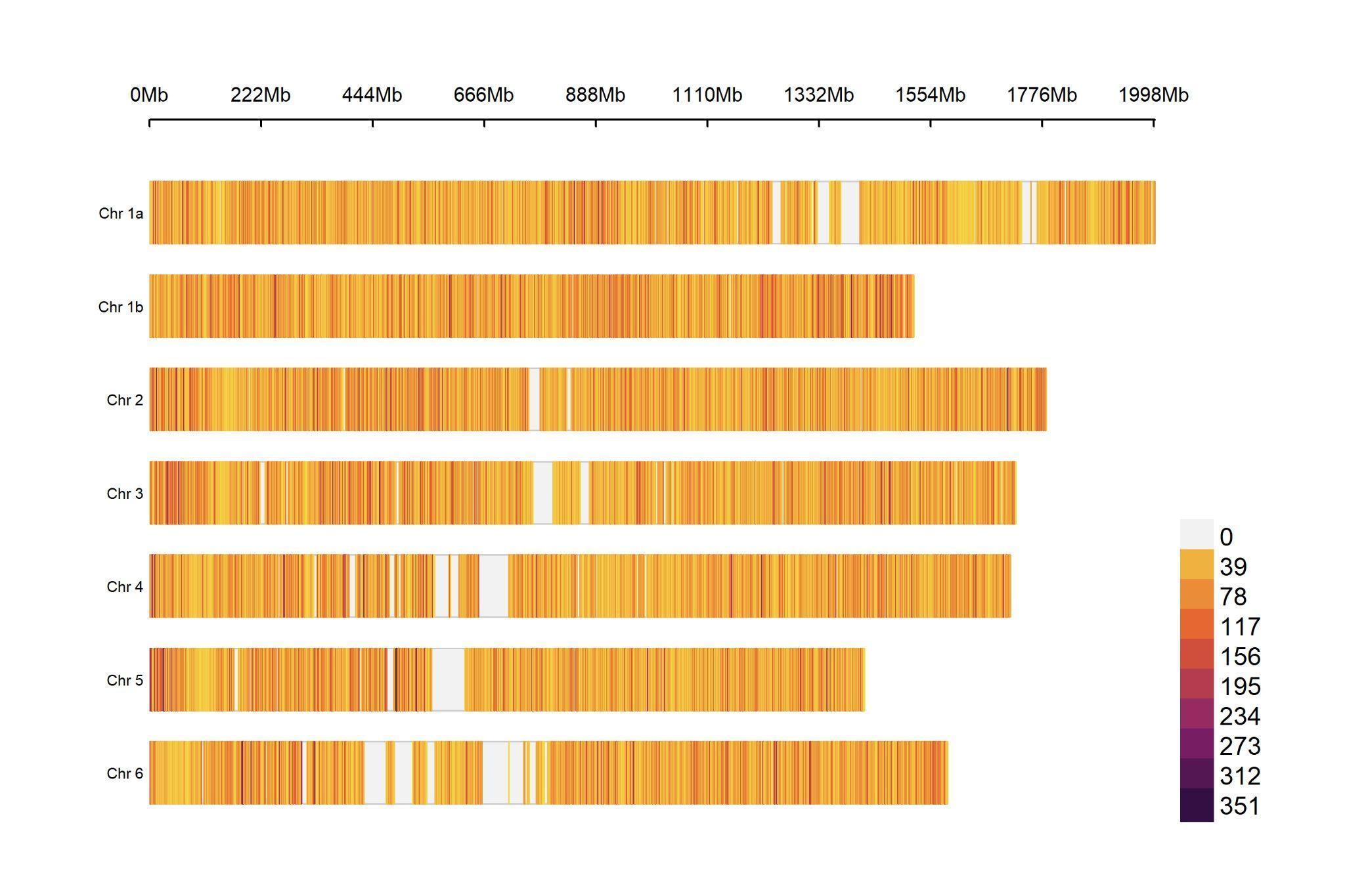
**

**Fig. S1|** **SNP density along the seven faba bean chromosomes.** The genotyping matrix contains 540 K variants obtained by combining SNPs from SPET (Single Primer Enrichment Technology) and GBS (Genotyping-by-sequencing) technologies. Bins in the heatmap are collared according to the number of SNPs per 1 mega base.


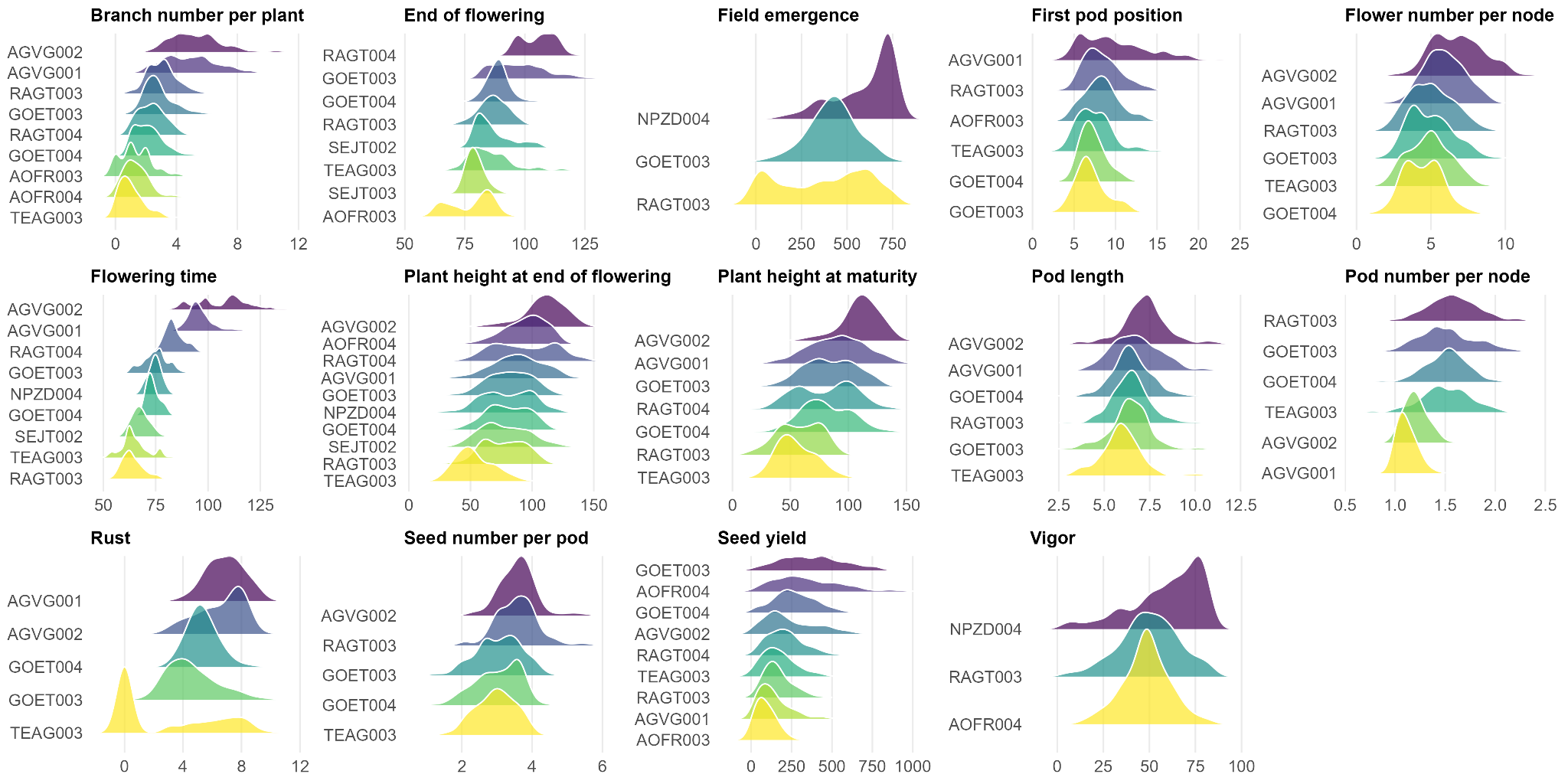


**Fig. S2 | Density distributions of trait values across environments for 14 agronomic traits.** Each ridge plot shows the distribution of individual trait values (adjusted means) across environments, ordered by mean score within each trait. The units of measurement for each trait are as follows: Branch number per plant, Flower number per node, and Pod number per node are measured in counts; End of flowering, Flowering time, and Rust disease score are measured in days and score, respectively; First pod position, Plant height at end of flowering, Plant height at maturity, and Pod length are measured in centimeters; Seed yield is measured in g/m²; and Vigor is measured as a visual score. Trait values were transformed as follows: Field emergence (cubed), Pod number per node (square root), and Vigor (squared).


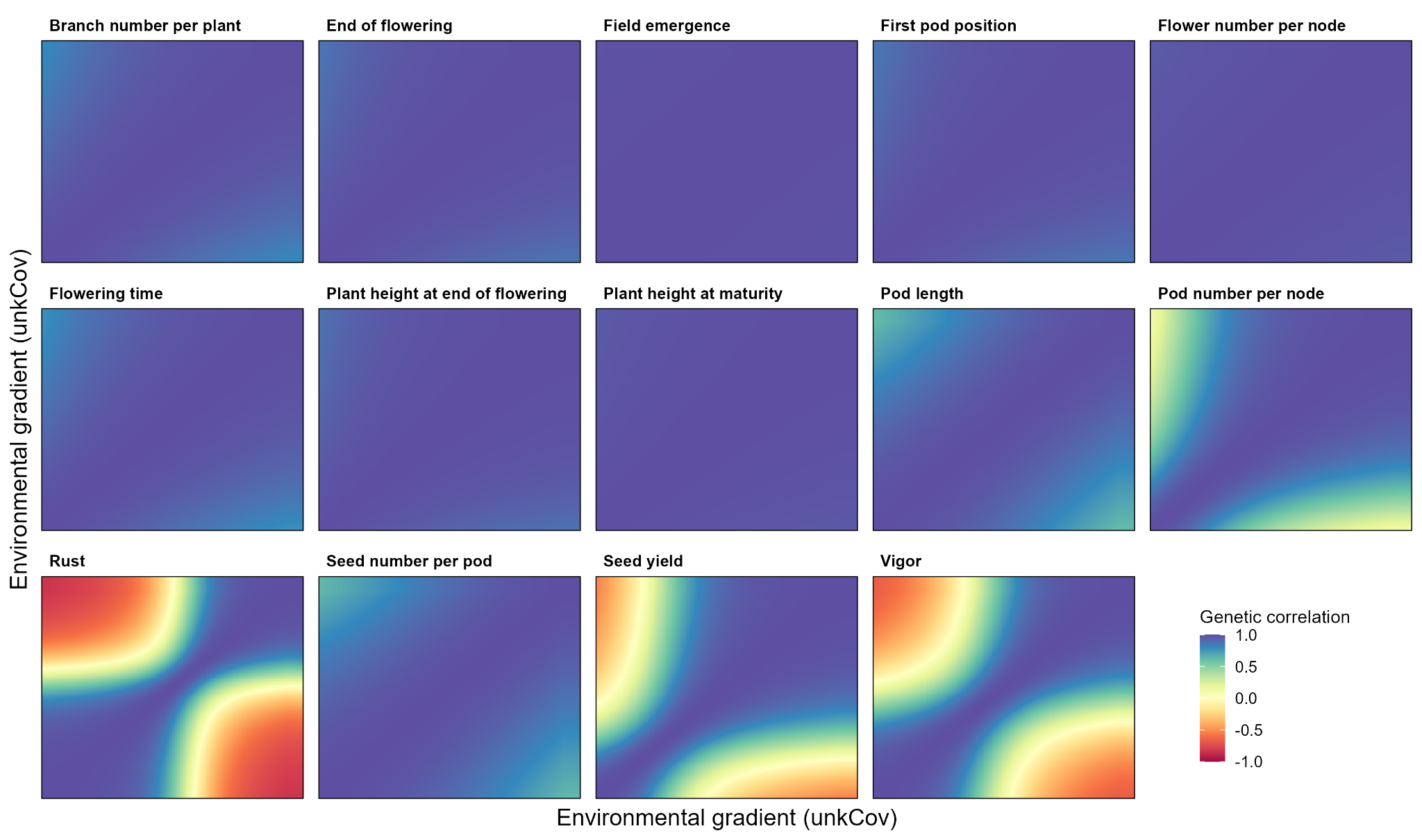


**Fig. S3|** **Estimated genetic correlations of 14 complex faba bean traits.** Genetic variances and covariances, which were used to calculate the correlations, were estimated using a Bayesian reaction norm model with an unknown covariate. Genotype-by-environment interaction is demonstrated by a decrease in genetic correlation as environments become more distant along the environmental gradient index.


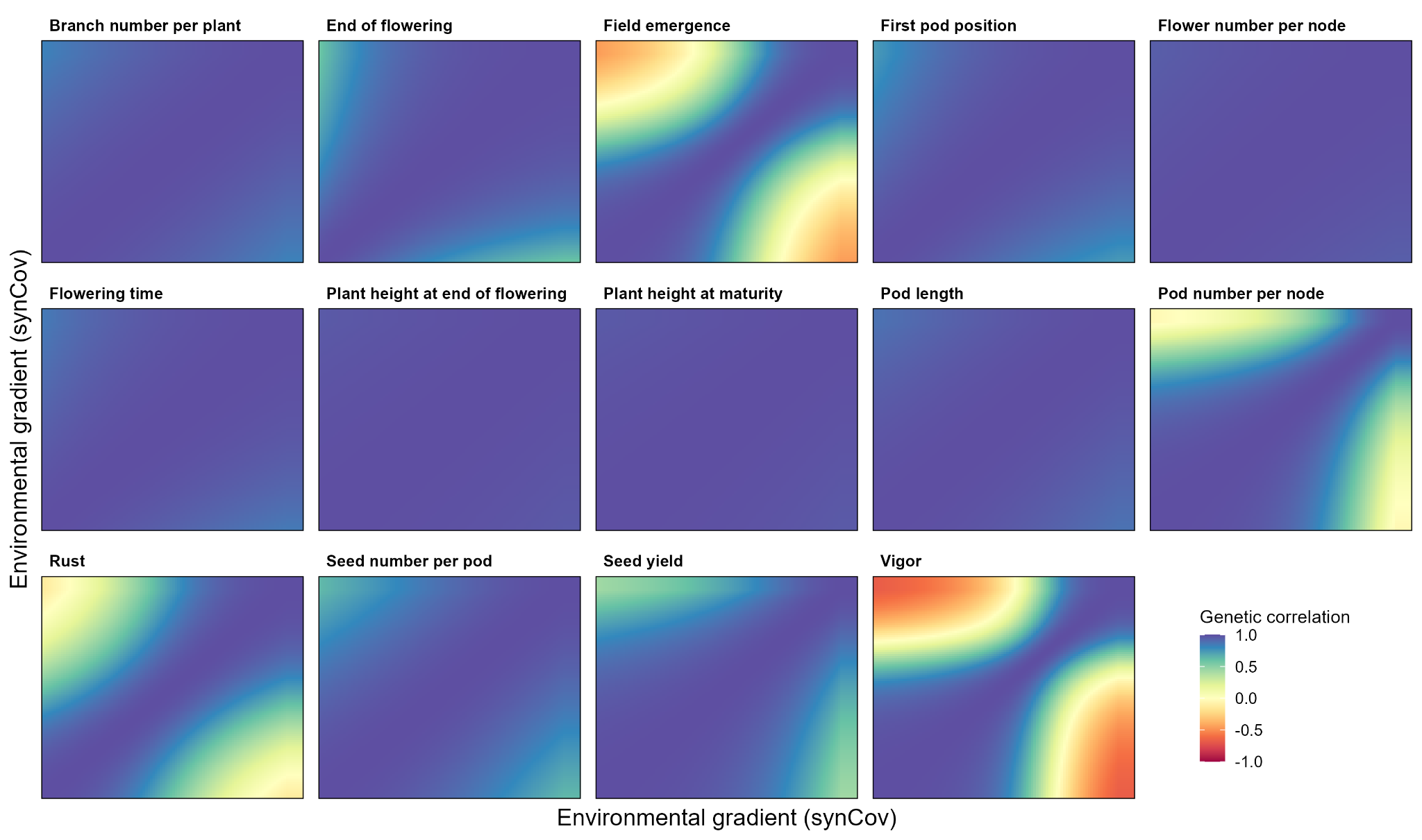


**Fig. S4|** **Estimated genetic correlations of 14 complex faba bean traits.** Genetic variances and covariances, which were used to calculate the correlations, were estimated using a Bayesian reaction norm model with a synthetic covariate. This covariate represents linear combinations of weather-derived covariables. Genotype-by-environment interaction is demonstrated by a decrease in genetic correlation as environments become more distant along the environmental gradient index.


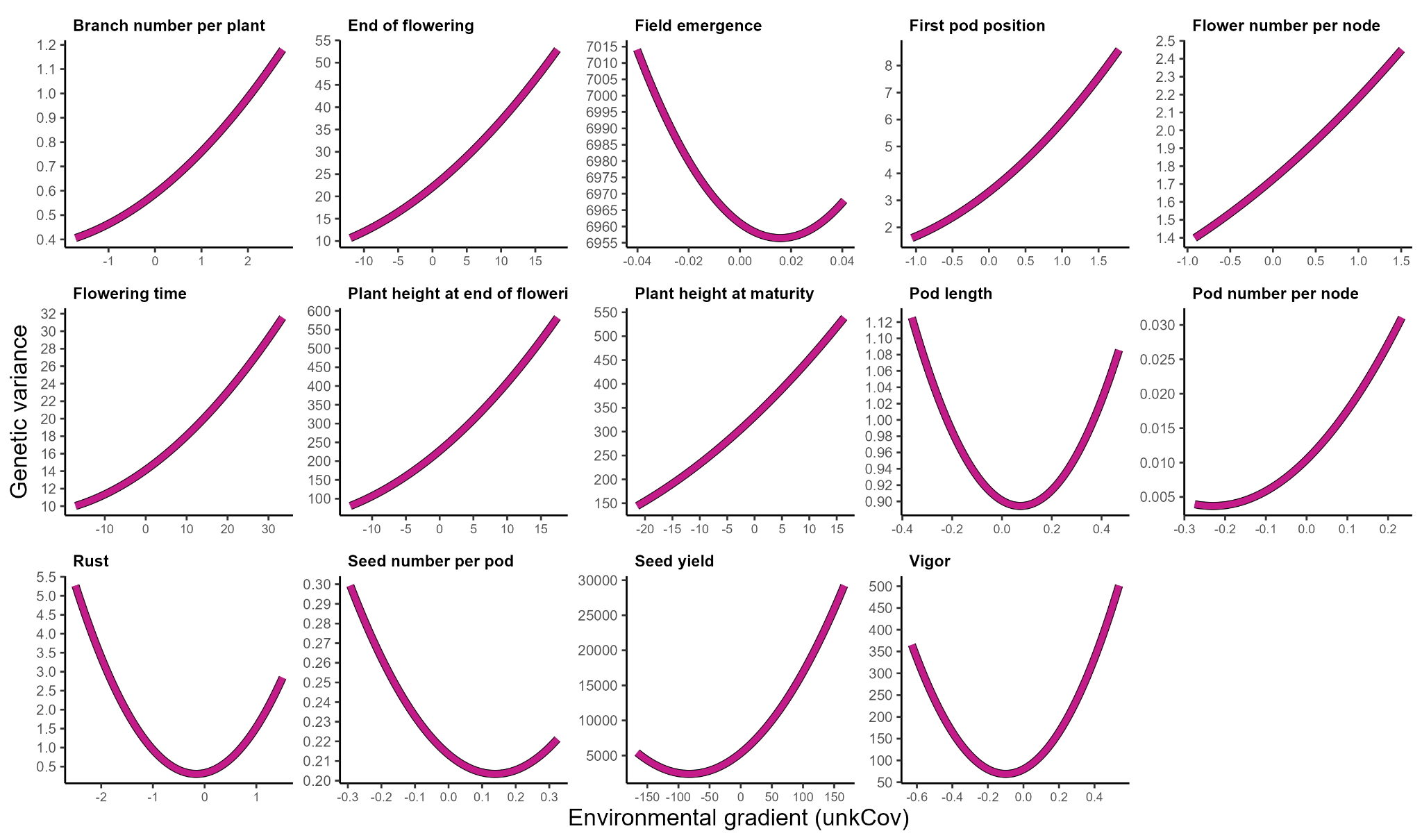


**Fig. S5|** **Genetic variance along the environmental gradient for 14 faba bean traits.** Genetic variances were estimated by fitting a Bayesian reaction norm model with an unknown covariate representing the environmental gradient. The unknown covariate is iteratively estimated during the model fitting process and represents solutions of the random environment main effect.


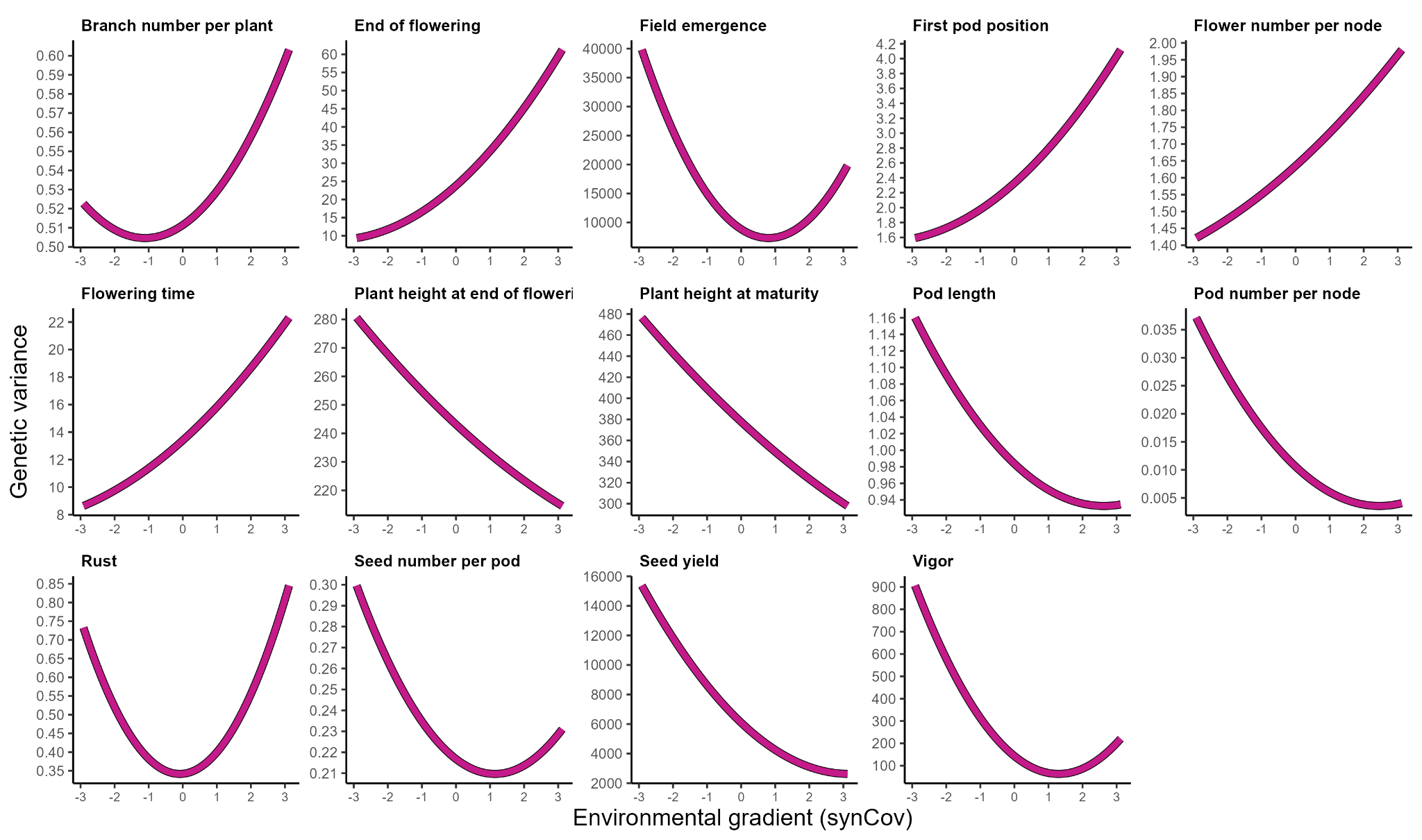


**Fig. S6|** **Genetic variance along the environmental gradient defined as by a synthetic covariate.** Genetic variances were estimated by fitting a Bayesian reaction norm model with a synthetic covariate representing the environmental gradient. This covariate is obtained by linear combinations of environmental variables e.g., temperature, precipitation, and radiation.


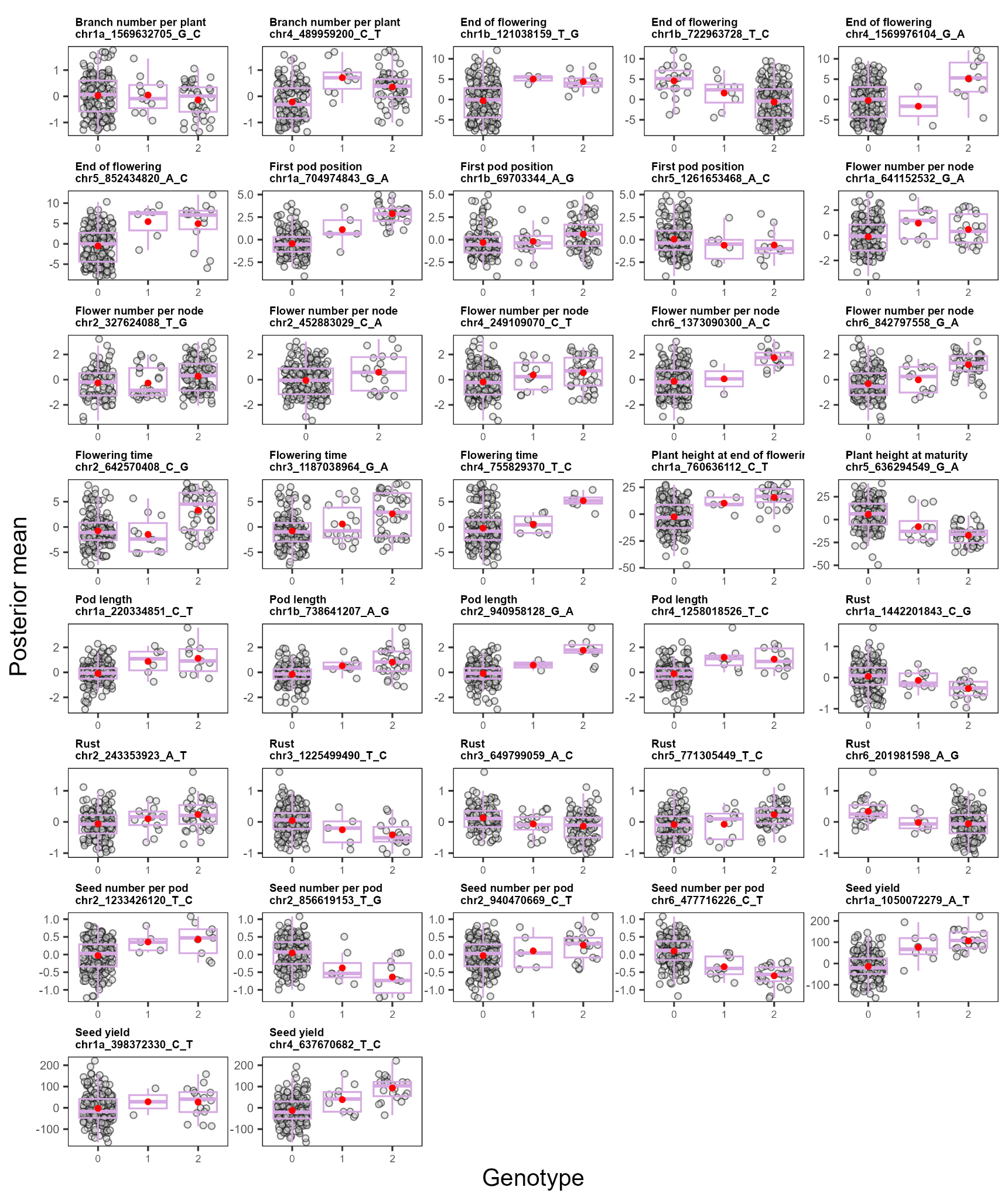


**Fig. S7|** **Distribution of intercept values according to allele state for 37 significant SNP marker-trait associations detected using multiple GWAS models.** The allele coding system counts the number of copies of the reference allele. Red dots depict the mean intercept value for the genotype class. The minor allele frequency threshold was 0.05.


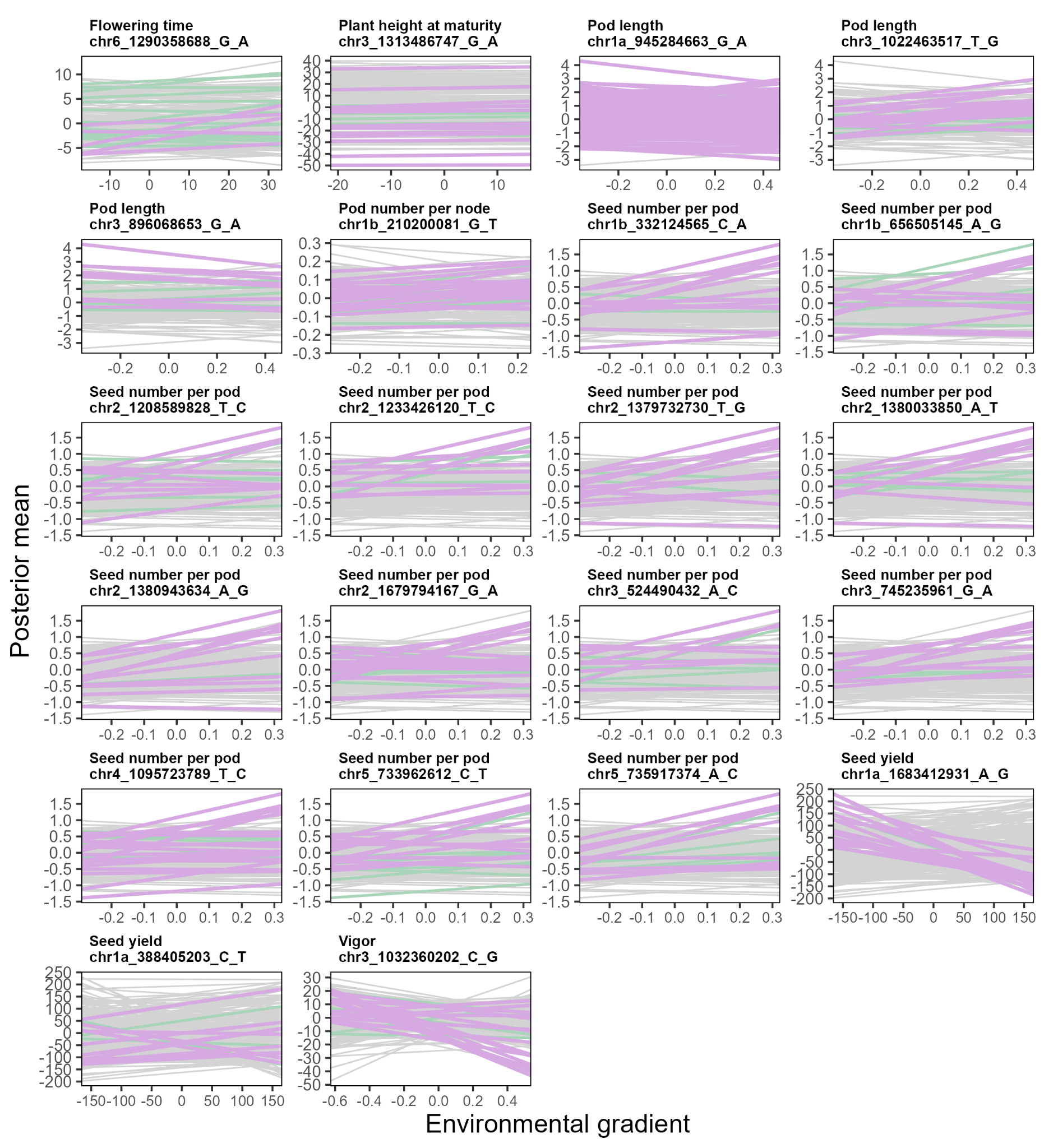


**Fig. S8|** **Scale-corrected reaction norms of 222 inbred faba bean lines for 22 unique marker-trait associations detected using multiple GWAS models.** GWAS was conducted on slope solutions of a Bayesian reaction norm model with an unknown covariate, which was estimated simultaneously from the data. Reaction norm lines are colored according to the allele state: purple lines represent homozygous genotypes for the alternative allele, green lines represent heterozygous genotypes, and gray lines represent homozygous genotypes for the reference allele. The minor allele frequency threshold was 0.05.


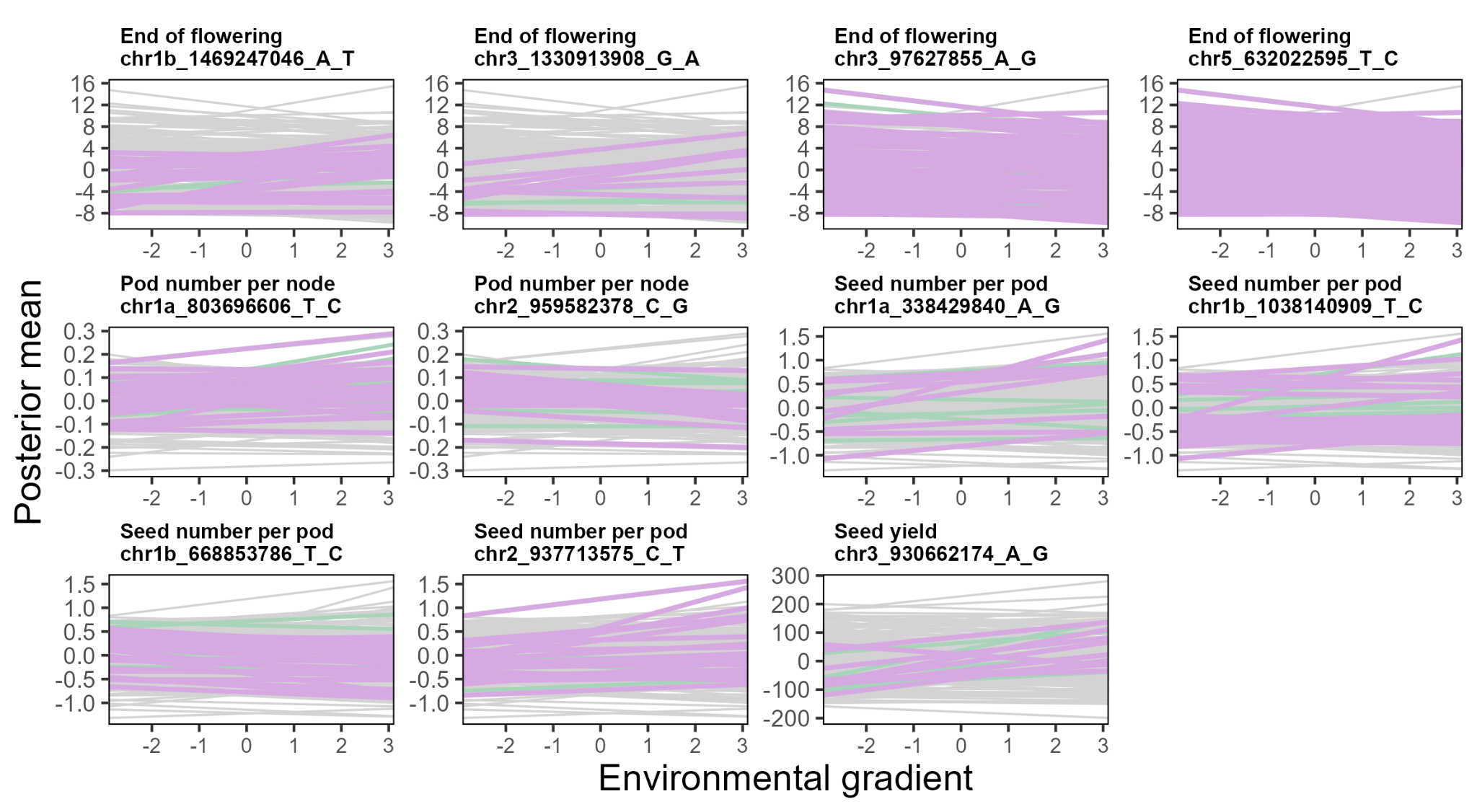


**Fig. S9|** **Scale-corrected reaction norms of 222 inbred faba bean lines for 11 unique marker-trait associations detected using multiple GWAS models.** GWAS was conducted on slope solutions of a Bayesian reaction norm model which used a covariate constructed as a linear combination of weather variables. Reaction norm lines are colored according to the allele state: purple lines represent homozygous genotypes for the alternative allele, green lines represent heterozygous genotypes, and gray lines represent homozygous genotypes for the reference allele. The minor allele frequency threshold was 0.05.


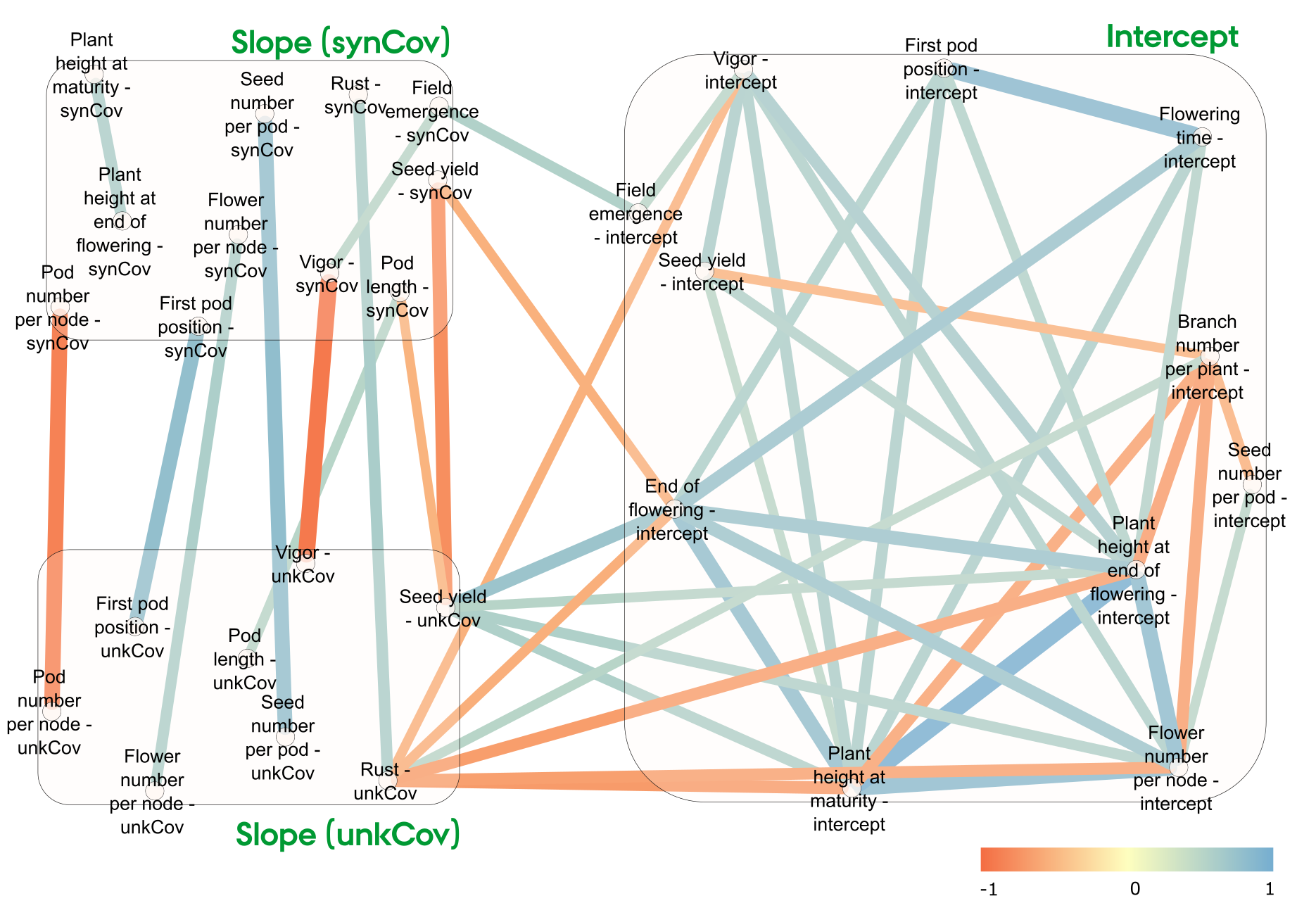


**Fig. S10|** **Network showing correlations between and within three latent variables estimated using reaction norm models for 14 faba bean traits.** Edges connecting nodes are shown for Pearson correlation values above |0.5|. Color gradient from red to blue and edge width map negative to positive correlation coefficients. Latent variables intercept and slope (unkCov) were obtained from a reaction norm model using an unknown covariate while solutions of slope (synCov) were obtained from a reaction norm model fitting a synthetic covariate.


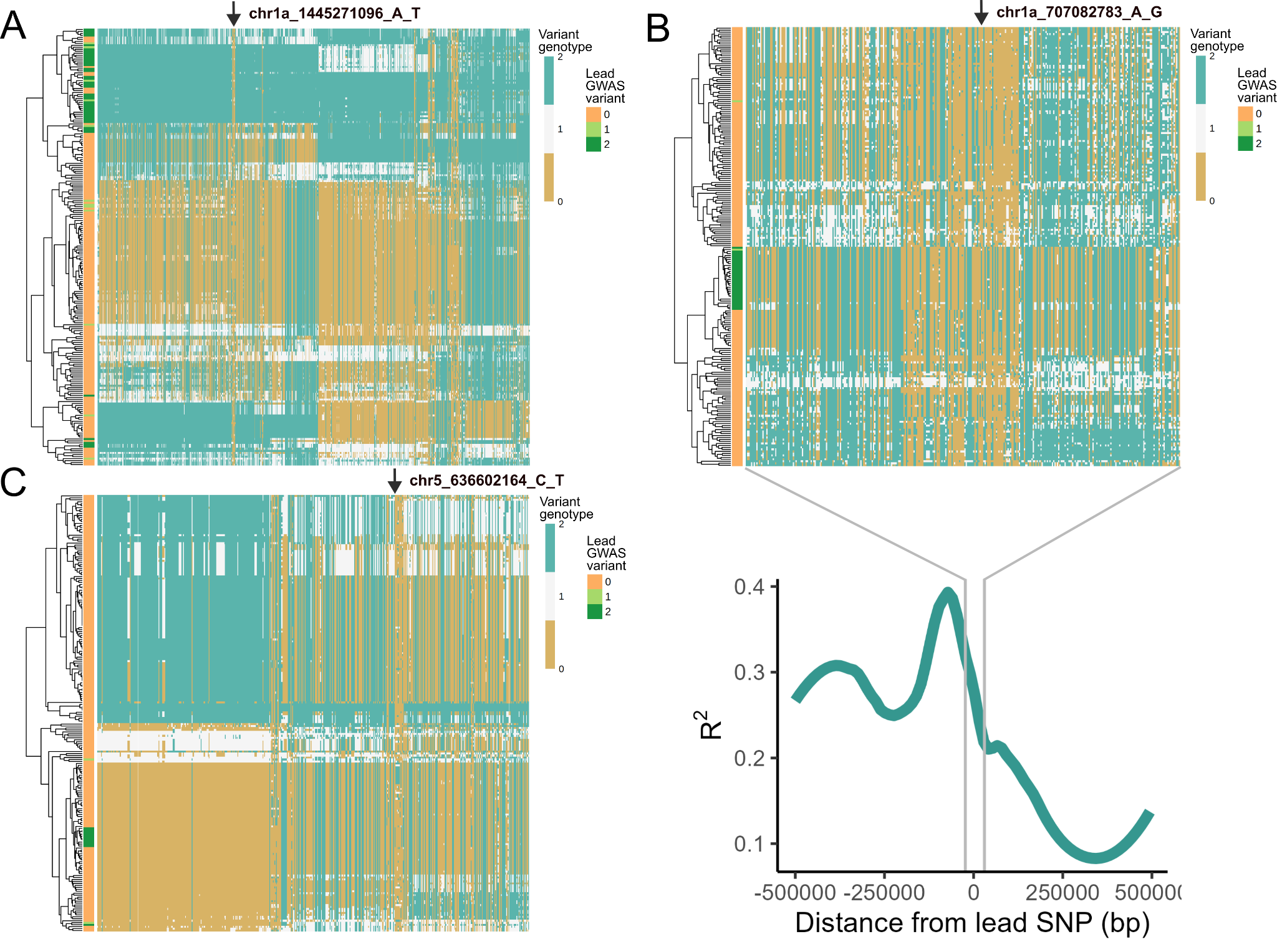


**Fig. S11 | Heatmaps of genotypic variation across genomic regions centered on lead GWAS variants.** Genomic regions surrounding each lead variant (±40.08 kb) were imputed using whole-genome resequencing (WGS) data as a reference. **A**, Heatmap for set ID 114 (Flowering time; **Supplementary Table S3**), where the variant with the lowest p-value from the GEMMA-LMM analysis is designated as the lead variant. **B**, Heatmap for SNP_ID 7 (Seed yield; **Supplementary Table S1**), where the GG haplotype is associated with the winter type of faba bean. The line plot uses WGS data to show the linkage disequilibrium (LD; R²) between the lead SNP (chr1a_707082783_A_G) and surrounding variants within a 1 Mb region. The LOESS curve (span = 0.4) is used to smooth the LD values, revealing the extent of the region enriched with highly correlated variants. Only two genes are annotated in the region (*Vfaba.Hedin2.R2.1g002126* and *Vfaba.Hedin2.R2.1g002127*). **C**, Heatmap for SNP_ID 32 (Plant height at end of flowering; **Supplementary Table S1**).

**
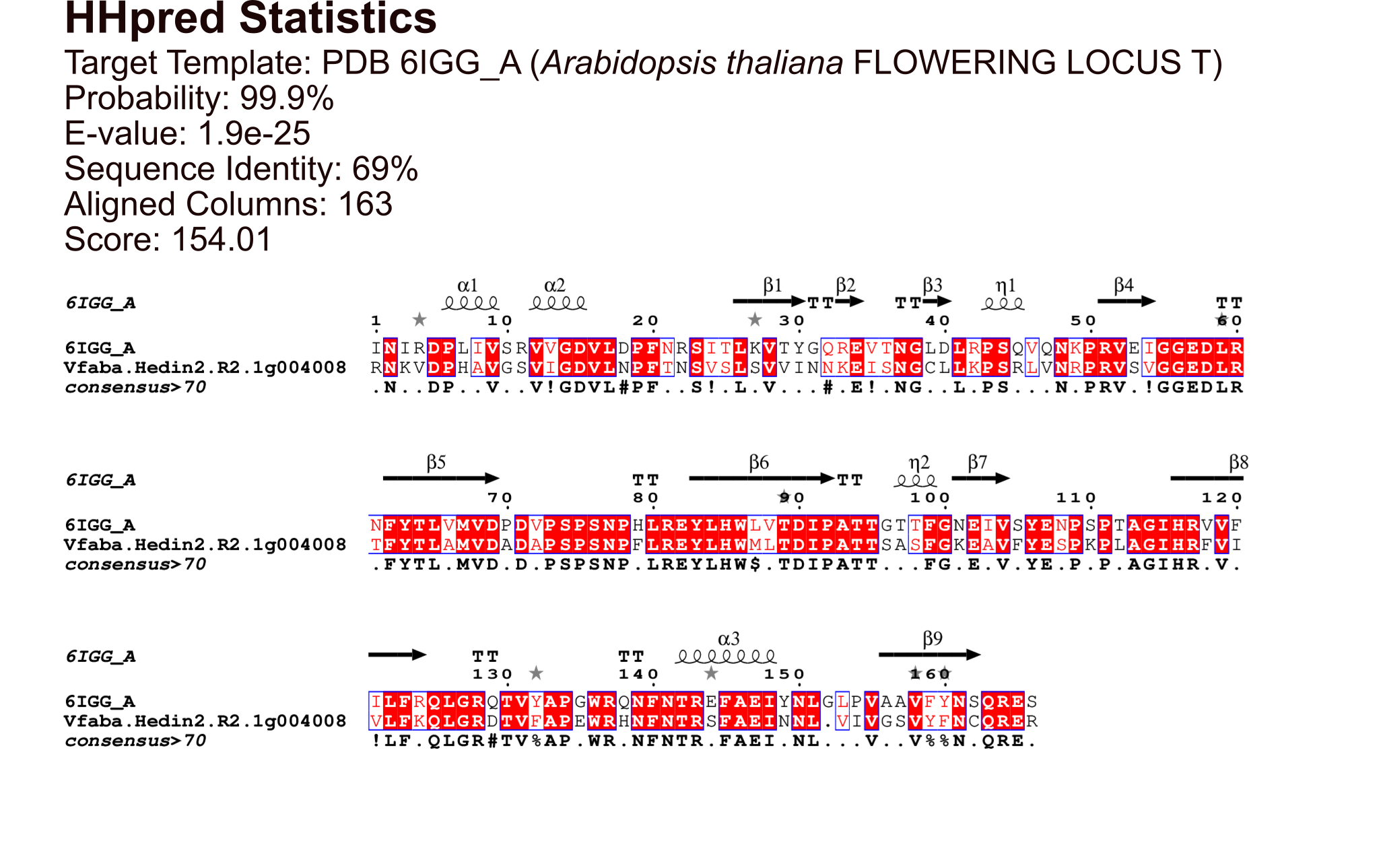
Fig. S12 | Sequence and structural alignment of *Vfaba.Hedin2.R2.1g004008* with Arabidopsis thaliana FLOWERING LOCUS T.** Profile-profile alignment generated by HHpred indicating high structural homology between the *Vicia faba* query sequence (*Vfaba.Hedin2.R2.1g004008*) and the *Arabidopsis thaliana* FLOWERING LOCUS T protein (PDB: 6IGG, chain A). The inset text at the top summarizes the HHpred validation statistics. The structural sequence alignment was rendered using ESPript 3.0. Strictly conserved residues are highlighted with white text on a solid red background. Residues with a similarity score greater than the global threshold are rendered as red text within blue frames. A consensus sequence (based on a >70% threshold) is shown below the alignment.


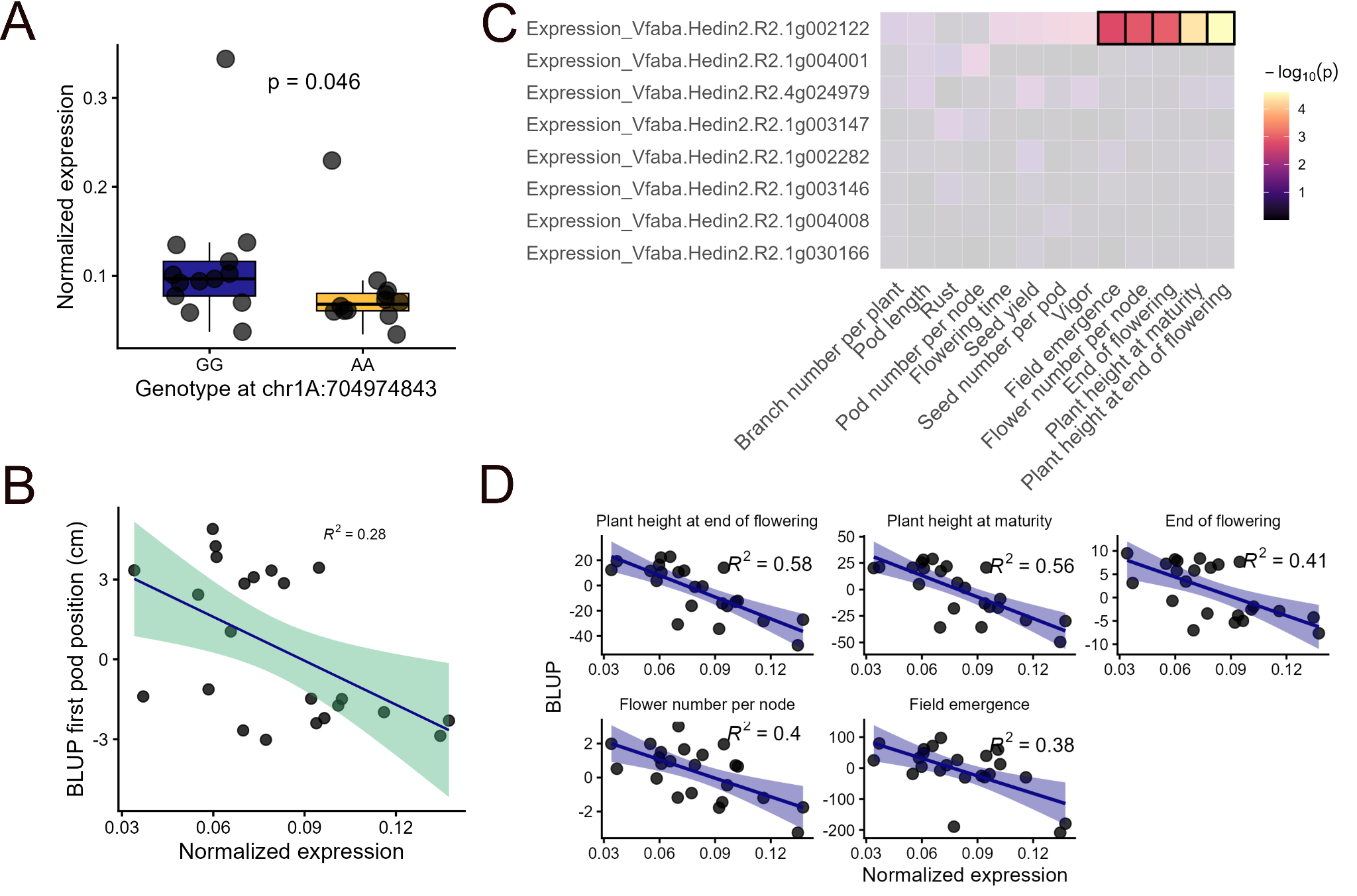


**Figure S13 | Regression of phenotypic traits on gene expression measured by RT-qPCR identifies a significantly associated gene. A**, Relative expression of *Vfaba.Hedin2.R2.1g002122* stratified by genotype at the genomic variant chr1A:704974843 (G/A) identified by GWAS for first pod position. Statistical significance between genotypes was assessed using a two-sided Wilcoxon rank-sum test (p = 0.046). Outliers (including the two extreme values shown) were removed prior to downstream analyses using Tukey’s 1.5× IQR rule. **B**, Linear regression between first pod position (BLUPs) and normalized expression of *Vfaba.Hedin2.R2.1g002122*. The association is significant at P < 0.01 (R² = 0.28). **C**, Heatmap showing significance levels (−log10 P-values) for pairwise associations between gene expression (RT-qPCR) and phenotypic traits. Each cell represents a linear regression test between one gene and one trait. Highlighted tiles indicate associations that remain significant after false discovery rate (FDR) correction (FDR < 0.05). **D**, Scatter plots of significant gene–trait associations retained after FDR correction. Linear regression fits are shown with 95% confidence intervals. Reported R² values indicate model fit for each association. Traits include plant height at end of flowering, plant height at maturity, end of flowering timing, flower number per node, and field emergence. Expression values correspond to normalized RT-qPCR measurements of *Vfaba.Hedin2.R2.1g002122*.
