# Supplementary material for: Dissecting the genetic basis of yield stability in faba bean by multi-environment analysis
Source: Genome Biol. 2026 Jul 20;27:231. doi: 10.1186/s13059-026-04181-0 (PMC13383516; doi:10.1186/s13059-026-04181-0)
Supplement: Supplementary file 3 — Additional file 3: Extended data. Contains Table S1 and Figs. S1 and S2. Table S1. Posterior estimates for genetic parameters. Fig. S1. Reaction norms. Fig. S2. Model comparison via cross-validation [file 13059_2026_4181_MOESM3_ESM.docx]

**Extended Data**

**Table S1|** Posterior estimates of SNP heritability (PVE), proportion of PVE explained by SNPs with sparse effect (PGE), and the putative number of large effect SNPs obtained from the Bayesian Sparse Linear Mixed Model (BSLMM).

| **Trait** | **Reaction norm-derived latent variables** | | | | | | | | |
| --- | --- | --- | --- | --- | --- | --- | --- | --- | --- |
|  | **Intercept** | | | **Slope (unkCov)** | | | **Slope (synCov)** | | |
|  | **PVE (SD)** | **PGE (SD)** | **SNPs (SD)** | **PVE (SD)** | **PGE (SD)** | **SNPs (SD)** | **PVE (SD)** | **PGE (SD)** | **SNPs (SD)** |
| Branch number per plant | 0.97 (0.03) | 0.21 (0.22) | 20 (22) | 0.48 (0.16) | 0.40 (0.30) | 37 (57) | 0.28 (0.12) | 0.41 (0.30) | 57 (70) |
| End of flowering | 0.99 (0.01) | 0.80 (0.23) | 83 (37) | 0.44 (0.14) | 0.40 (0.30) | 101 (86) | 0.59 (0.15) | 0.68 (0.23) | 22 (17) |
| Field emergence | 0.77 (0.11) | 0.52 (0.31) | 89 (68) | 0.25 (0.14) | 0.40 (0.30) | 30 (36) | 0.58 (0.22) | 0.38 (0.29) | 65 (76) |
| First pod position | 0.93 (0.05) | 0.59 (0.21) | 17 (16) | 0.22 (0.14) | 0.40 (0.30) | 71 (92) | 0.14 (0.10) | 0.42 (0.31) | 35 (53) |
| Flower number per node | 0.99 (0.01) | 0.57 (0.31) | 47 (26) | 0.23 (0.18) | 0.40 (0.30) | 21 (32) | 0.28 (0.15) | 0.40 (0.30) | 26 (39) |
| Flowering time | 0.98 (0.02) | 0.71 (0.25) | 48 (33) | 0.78 (0.10) | 0.49 (0.31) | 131 (104) | 0.32 (0.19) | 0.42 (0.30) | 49 (73) |
| Plant height end of flowering | 0.94 (0.03) | 0.83 (0.16) | 110 (67) | 0.47 (0.13) | 0.45 (0.30) | 40 (59) | 0.51 (0.16) | 0.33 (0.28) | 52 (72) |
| Plant height at maturity | 0.98 (0.04) | 0.80 (0.20) | 49 (22) | 0.32 (0.15) | 0.39 (0.29) | 32 (54) | 0.47 (0.17) | 0.41 (0.29) | 39 (42) |
| Pod length | 0.99 (0.02) | 0.42 (0.23) | 25 (18) | 0.83 (0.08) | 0.40 (0.29) | 111 (94) | 0.81 (0.09) | 0.30 (0.28) | 69 (73) |
| Pod number per node | 0.87 (0.08) | 0.29 (0.25) | 78 (81) | 0.63 (0.12) | 0.32 (0.26) | 74 (75) | 0.65 (0.11) | 0.33 (0.28) | 63 (80) |
| Rust | 0.78 (0.08) | 0.42 (0.31) | 130 (90) | 0.92 (0.05) | 0.25 (0.25) | 51 (57) | 0.60 (0.08) | 0.38 (0.30) | 26 (28) |
| Seed number per pod | 0.96 (0.05) | 0.44 (0.24) | 33 (28) | 0.95 (0.05) | 0.52 (0.21) | 11 (9) | 0.79 (0.12) | 0.55 (0.23) | 22 (30) |
| Seed yield | 0.89 (0.04) | 0.66 (0.29) | 137 (70) | 0.89 (0.04) | 0.71 (0.23) | 49 (58) | 0.78 (0.09) | 0.52 (0.31) | 48 (45) |
| Vigor | 0.70 (0.08) | 0.55 (0.33) | 69 (66) | 0.50 (0.13) | 0.51 (0.29) | 32 (44) | 0.50 (0.15) | 0.37 (0.29) | 84 (100) |

Posterior means and standard deviations (SD) of genetic parameters were estimated using 120,000 MCMC samples. We fitted two reaction norm models (RNM1 and RNM2) that primarily differ in the environmental quality index used. RNM1 internally estimates an unknown covariate (unkCov), whereas RNM2 uses a synthetic covariate (synCov) constructed as a linear combination of environmental variables.


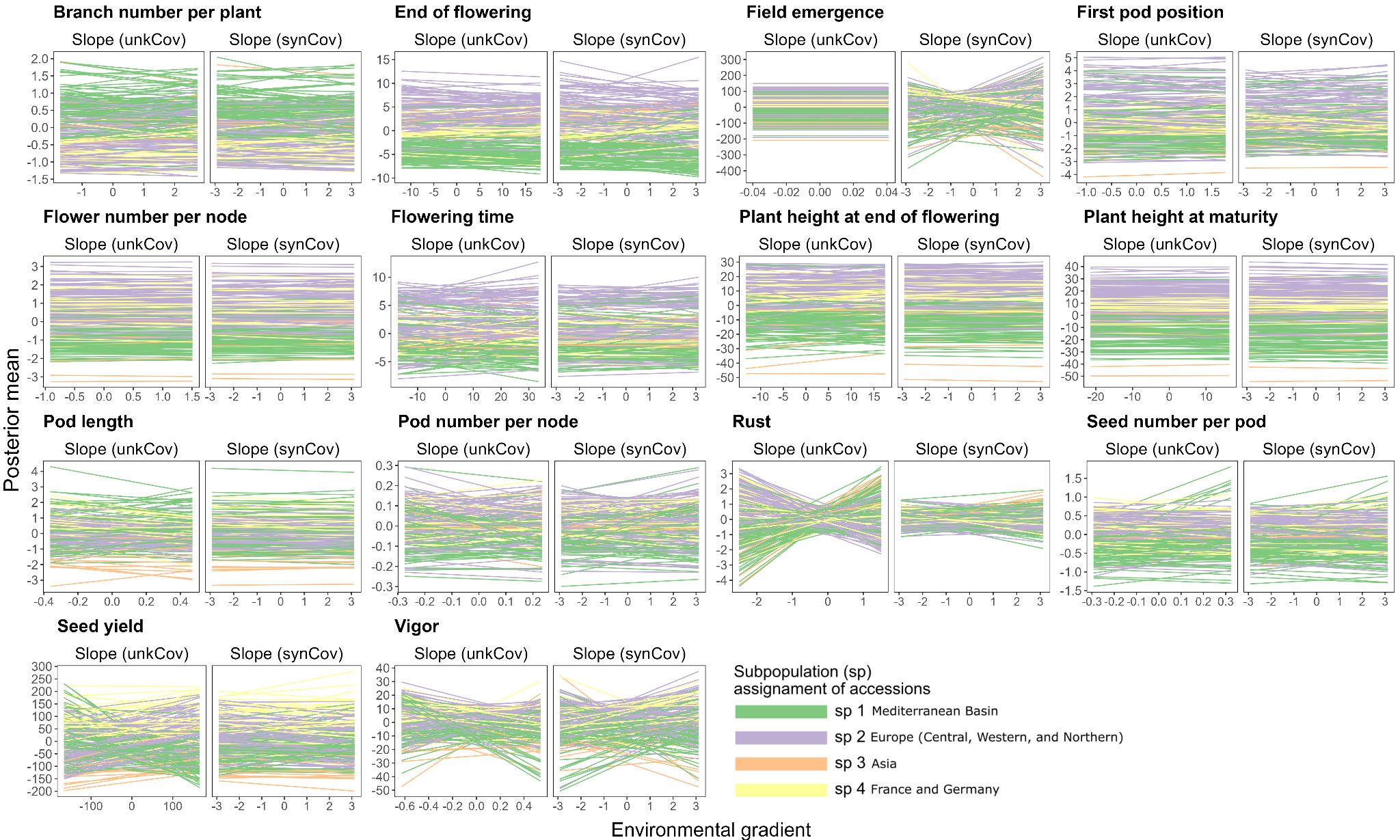


**Fig. S1|** **Scale-corrected reaction norms for 222 accessions of faba bean**. Accessions are grouped by four colors based on population structure inferred via ADMIXTURE analysis (see **Fig. 1A**). Each trait's reaction norms are shown for two variations of a reaction norm model, differing by the environmental covariate: an unknown covariate (unkCov) and a synthetic covariate derived from environmental variables (synCov).


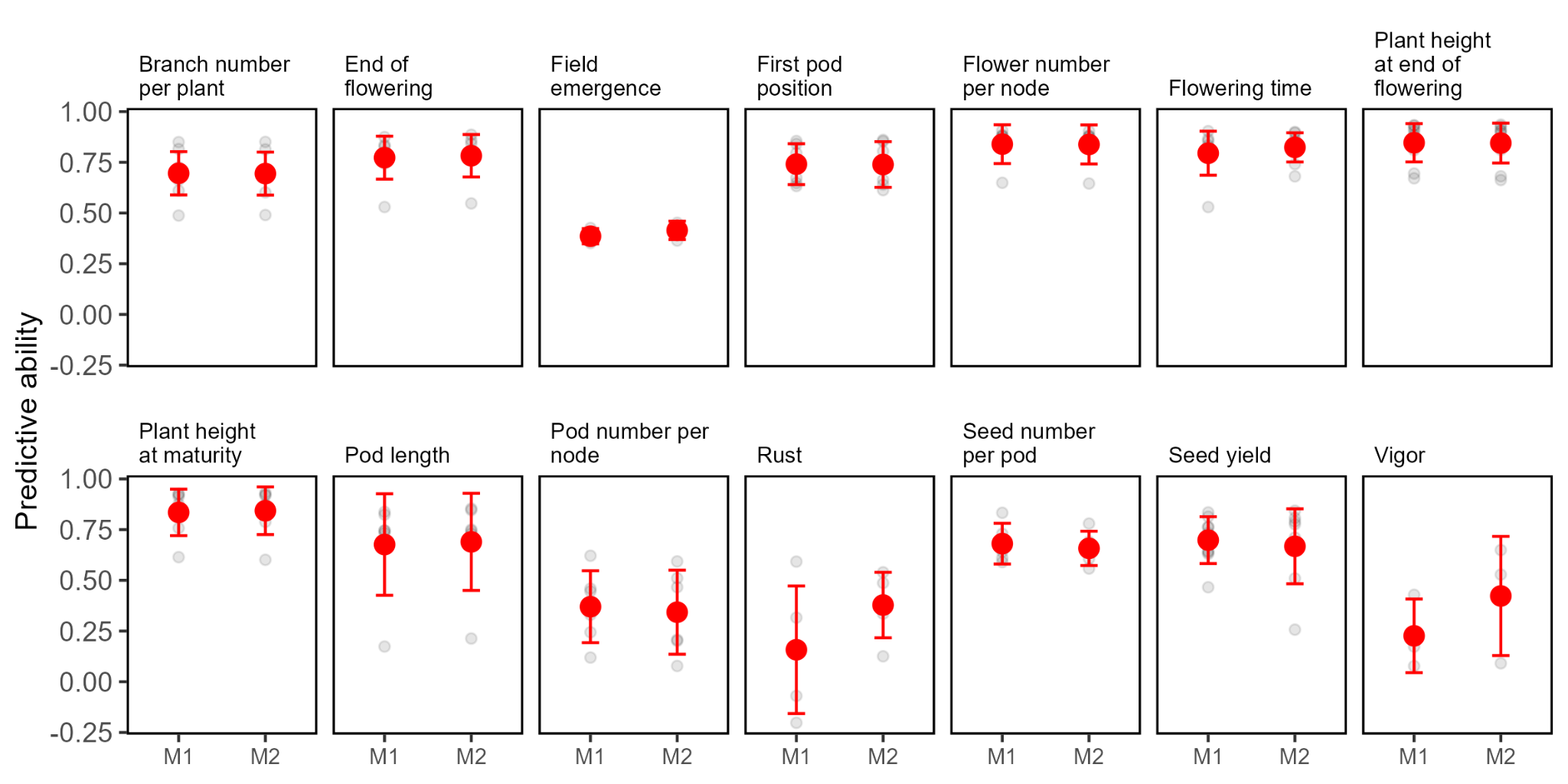


**Fig. S2 | Comparison of two genomic prediction models using a leave-one-environment-out cross-validation scheme.** In model M1, genotype-by-environment (G×E) interaction is modeled via a covariance structure obtained from the element-wise product of the genomic and environmental relationship matrices (Jarquin et al., 2014). In the proposed model M2, G×E is assessed using random regressions on two eigenvectors computed from environmental variables. Error bars represent the standard deviation of the correlations between predicted and observed performance in the left-out environment.
